# Evaluation of pathogenicity of *Salmonella* Gallinarum strains harbouring deletions in genes whose orthologues are conserved pseudogenes in *S*. Pullorum

**DOI:** 10.1371/journal.pone.0200585

**Published:** 2018-07-20

**Authors:** Diego Felipe Alves Batista, Oliveiro Caetano de Freitas Neto, Adriana Maria de Almeida, Grazieli Maboni, Tatiane Furtado de Carvalho, Thaynara Parente de Carvalho, Paul Andrew Barrow, Angelo Berchieri

**Affiliations:** 1 Post Graduate Program in Agricultural and Livestock Microbiology, Department of Veterinary Pathology, School of Agriculture and Veterinarian Sciences, São Paulo State University (Unesp), campus at Jaboticabal, São Paulo, Brazil; 2 Department of Veterinary Sciences, Federal University of Paraíba, Areia, Paraíba, Brazil; 3 School of Veterinary Medicine and Science, University of Nottingham, Sutton Bonington, Leicestershire, United Kingdom; 4 Department of Veterinary Clinic and Surgery, School of Veterinary, Federal University of Minas Gerais (UFMG), Belo Horizonte, Minas Gerais, Brazil; Universidad de la Republica Uruguay, URUGUAY

## Abstract

The diseases caused by *Salmonella* Gallinarum and *S*. Pullorum in chickens known as fowl typhoid and pullorum disease, respectively, pose a great threat to the poultry industry mainly in developing countries, since they have already been controlled in the developed ones. These bacteria are very similar at the genomic level but develop distinct host-pathogen relationships with chickens. Therefore, a deep understanding of the molecular mechanisms whereby *S*. Gallinarum and *S*. Pullorum interact with the host could lead to the development of new approaches to control and, perhaps, eradicate both diseases from the chicken flocks worldwide. Based on our previous study, it was hypothesised that metabolism-related pseudogenes, fixed in *S*. Pullorum genomes, could play a role in the distinct host-pathogen interaction with susceptible chickens. To test this idea, three genes (*idnT*, *idnO* and *ccmH*) of *S*. Gallinarum str. 287/91, which are pseudogenes on the *S*. Pullorum chromosomes, were inactivated by mutations. These genetically engineered strains grew well on the solid media without any colony morphology difference. In addition, similar growth curves were obtained by cultivation in M9 minimal medium containing D-gluconate as the sole carbon source. Infection of chickens with *idnTO* mutants led to increased numbers of bacteria in the livers and spleens at 5 days post-infection, but with slightly decreased heterophil infiltration in the spleens when compared to the wild-type strain. On the other hand, no significant phenotypic change was caused by mutation to *ccmH* genes. Apart from the above-mentioned alterations, all *S*. Gallinarum strains provoked similar infections, since mortality, clinical signs, macroscopic alterations and immune response were similar to the infected chickens. Therefore, according to the model applied to this study, mutation to the *idnTO* and *ccmH* genes showed minor impact on the fowl typhoid pathogenesis and so they may be relics from the ancestor genome. Our data hints at a more complex mechanism driving the distinct host-pathogen interaction of *S*. Gallinarum/Pullorum with chickens than differential inactivation of a few genes.

## Introduction

Fowl typhoid is a severe, systemic disease caused by *Salmonella enterica* subsp. *enterica* serovar Gallinarum biovar Gallinarum (*S*. Gallinarum) that occurs in some species of birds at any age [1]. Diseased animals show somnolence, droopy wings, ruffled feathers, anorexia and green-yellowish diarrhoea, and mortality can be as high as 100% [1,2], though it hardly achieves this proportion in the field conditions [3]. Pullorum disease provoked by *Salmonella enterica* subsp. *enterica* serovar Gallinarum biovar Pullorum (*S*. Pullorum) manifests as an acute disease mainly in very young birds, which present clinical signs resembling fowl typhoid but with fluid, white diarrhoea [4,5]. Convalescent birds may transmit *S*. Pullorum vertically to the progeny [4,6].

Neither *S*. Gallinarum nor *S*. Pullorum evoke intense pro-inflammatory host responses [7,8], thus bacteria are able to invade the host tissues by stealth from the intestines. Supporting this idea, Setta et al. [9] found that epithelial chicken kidney cell (CKC) transcribed low levels of CXCLi1, CXCLi2 and IL6 cytokines to infections with *S*. Gallinarum and *S*. Pullorum. By reaching the systemic sites, these bacteria are recognised by the antigen-presenting cells (APC) and then an adaptive immunity is developed. The nature of this response to *S*. Gallinarum is as yet poorly understood, nevertheless *S*. Pullorum infection induces a response more closely resembling a Th2-type with low IFNγ and high IL-4 expression, contrasting to the Th1-like response stimulated by *S*. Enteritidis and characterised by high levels of splenic IFNγ and IL-18 [10].

Knowledge about *S*. Gallinarum and *S*. Pullorum genetics and how it relates to their pathogenicity is still under construction. These bacteria have arisen separately from a common ancestor [11], which was recently shown to be from a lineage of *S*. Enteritidis called “second clade” [12]. The genomic comparison performed by Langridge et al. [12] identified 231 and 212 conserved pseudogenes amongst the *S*. Gallinarum and *S*. Pullorum genomes analysed, respectively. Such high numbers are compatible with an evolution based on a gradual loss of protein-coding capacity through accumulation of mutations.

It has been shown that some of the genes involved in the intestinal colonisation and survival of the broad-host-range *Salmonella* serovars, such as *cbi*, *pdu* and *ttr*, harbour deleterious mutations in *S*. Gallinarum and *S*. Pullorum genomes [13,14] supposedly as a consequence of these organisms not having the intestines as a central feature of their infections. Genomic comparisons performed amongst some *S*. Gallinarum and *S*. Pullorum genomes have revealed a small number of conserved, biovar-specific pseudogenes [15,16], some of which checked and confirmed by PCR [16]. Additionally, it would appear that inactivation of anaerobic metabolic pathways, as exemplified by disruption of *ccm* (c-type cytochrome maturation) and *tor* (trimethylamine N-oxide reductase) genes, is more a feature of *S*. Pullorum genomes than of *S*. Gallinarum [16]. Thus, we hypothesised that inactivation of metabolism-related genes could lead to a distinct pattern of virulence gene regulation resulting in different diseases.

There is a growing body of evidence towards the linkage between the metabolism of a given pathogen and its virulence [17,18,19,20,21, 22]. It was shown by Njoroge et al. [18] that, in a glucose-dependent manner, the positive regulators Cra and KdpE bind to the promoter region of *ler* genes leading to expression of the locus of enterocyte effacement (LEE) genes of enterohemorragic *Escherichia coli* (EHEC). Glucose and glycolysis were shown by Bowden et al. [17] to play important roles in *S*. Typhimurium survival within RAW264.7 macrophages and in the expression of its full pathogenicity for BALB/c mice. A more recent study, conducted by Jelsbak et al. [21] also demonstrated that deletions of the *purN* and / or *purT* genes, which play roles in the purine biosynthesis pathway, impaired *S*. Typhimurium pathogenicity *in vivo*.

Based on the above-presented evidences, we tested in this study our hypothesis that some conserved biovar-specific pseudogenes, virtually present in every *S*. Gallinarum or *S*. Pullorum and predicted to be involved in the bacterial metabolism, would have an impact on the bacterium virulence and on its interaction with the host. The *idnT-idnO* (*idnTO*) and *ccmH* genes of *S*. Gallinarum were targeted since their nucleotide sequences have no deleterious mutation [16]. The *idn* operon, named after its importance for L-idonate metabolism, plays a role in a subsidiary system (GntII) for gluconate usage based on an *E*. *coli* model [23]. IdnT protein was shown to transport L-idonate [23] and D-gluconate [24] at high affinity, which are metabolised through the Entner-Doudoroff pathway [23] in order to provide carbon source and energy to the bacterial cell. A study in which J774-A.1 mouse cells were infected with *S*. Typhimurium SL1344 raised evidences that D-gluconate along with other related carbohydrates may be the main carbon sources to support the intracellular growth of *Salmonella* [25].

The *ccm* operon encodes for proteins responsible for maturation of a c-type cytochrome (ccm—cytochrome c maturation), which in *E*. *coli* is chiefly expressed under anaerobiosis [26, 27]. The *ccmH* gene (previously named as *yejP*) was firstly shown by Grove et al. [26] to play an important role in cytochrome c maturation in *E*. *coli*. Later on, Tanapongpipat et al. [27] demonstrated that deletion of any *ccm* gene (A-H) could block c-cytochrome maturation. Thus, in the present study we aimed to investigate the effects of mutation to the genes *idnTO* and *ccmH* of *S*. Gallinarum as their orthologues are conserved pseudogenes in *S*. Pullorum genomes. The phenotype of the genetically engineered strains was evaluated *in vitro* and by infection of susceptible chickens.

## Material and methods

### Ethical statements

Chicken challenge experiments were carried out in accordance with the recommendations in the Ethical Principles on Animal Experimentation (CEUA) of the National Council for the Control of Animal Experimentation (CONCEA). The protocol was approved by the Ethical Committee on Animal Experimentation from the School of Agriculture and Veterinarian Sciences on 03 February 2014 (Permit Number: 028358/13). Infected animals were observed on a daily-basis and those showing severe clinical signs of fowl typhoid, such as ruffled feathers, anorexia, somnolence and greenish diarrhoea, were humanely sacrificed by cervical dislocation performed by trained veterinarians. The genetically engineered organisms used in this study were constructed with permission granted by the National Technical Commission on Biosafety on 14 April 2015 (DOU—página 11, seção 1 de 14 de Abril de 2015 / permit number: 4.429/2015).

### Sequence analyses

The *idnT*, *idnO* and *ccmH* coding sequences (CDS) of *S*. Gallinarum str. 287/91 (AM933173.1), *S*. Gallinarum str. 9184 (CP019035.1), *S*. Pullorum str. CDC1983-67 (CP003786.1) and *S*. Pullorum str. RKS5078 (CP003047.1) were recovered from the GenBank database [28] as their genomes or chromosomes were completely sequenced and assembled. CDS were aligned by using the CLC Bio software version 7.7 for windows.

### Strains used and genetically engineered organisms

The spontaneous nalidixic acid-resistant *Salmonella* Gallinarum str. 287/91 (SG287/91) and *S*. Pullorum str. 449/87 (SP449/87) were used as positive controls of fowl typhoid and pullorum disease, respectively. SG287/91 also provided the genetic background for constructing the mutant strains because its genome has been completely sequenced [13] and its pathogenicity has already been characterised by our group [29,30]. Site-directed mutagenesis was performed according to Datsenko and Wanner [31] as follows: The antibiotic-resistance gene plus the FRT sites were amplified from the pKD3 (AY048742.1) or pKD4 (AY048743.1) plasmids through PCR by way of 70-bp hybrid primers. SG287/91 harbouring the red recombinase expression plasmid (pKD46) was cultured in Difco Lennox Lysogeny broth (LB—Becton, Dickinson and Company, US) containing 20 μg / mL ampicilin and 0.2 M L-arabinose (Sigma-Aldrich, US) at 28 °C under shaking (175 rpm) until the culture OD reached between 0.5 and 0.7. Transformation of the amplified fragments was performed through electroporation and positive clones were identified by PCR from the colonies grown on LB agar (Becton, Dickinson and Company, US) containing 20 μg / mL appropriate antibiotic (chloramphenicol or kanamycin).

Mutations to *idnTO* (SGΔ*idnTO*) and *ccmH* (SGΔ*ccmH*-1 and SGΔ*ccmH*-2) alleles were transduced to a clean genetic background (parental SG287/91) by P22 phage transduction, which was further used to construct the complete SGΔ*ccmH* and double (SGΔ*ccmHidnTO*) mutants. Chloramphenicol / kanamycin resistance genes were removed from the bacterial chromosomes prior to the animal experimentation. In short, the pCP20 plasmid, encoding for the FLP enzyme that targets the FRT sites, was transformed into the SG engineered strains by electroporation. After a few passages in LB broth at 42 °C (175 rpm), bacteria were streaked onto LB agar, with and without the antimicrobials here used, and those clones that had lost all the resistances introduced during the procedure were chosen. This clean genetic background was further confirmed by PCR. Mutant strains were checked as to roughness using 1:1000 diluted acriflavine and smooth bacteria were stored at -80 °C in Lysogeny broth supplemented with 30% glycerol. Primer sequences and details on the alterations introduced on SG287/91 chromosome are shown in the S1 and S2 Tables, respectively.

### *In vitro* studies

SG287/91 and its derivative engineered strains were cultured in 10 mL of LB broth for 24 hours at 37 °C. Next day, the cultures were streaked onto Brilliant Green agar (BGA) without antibiotics and colony morphology was assessed. In parallel, cultures were centrifuged at 3,000 x g for 10 minutes and suspended in 1 mL sterile PBS, pH 7.4 (10-fold concentrated). A volume of the concentrated cultures was inoculated in 10 mL of M9 minimal medium so that the initial OD_595_ was about 0.05 for every culture. M9 medium was prepared as described by Jelsbak et al. [21] saved for its supplementation, in this study, with 0.5% tryptone due to *S*. Gallinarum auxotrophy. D-Gluconic acid sodium salt (Sigma-Aldrich, US) was added as the sole carbon source at a 0.5% concentration. M9 cultures were incubated at 37 °C in a shaker incubator (150 rpm) and bacterial growth was monitored at 595 nm (OD_595_) in an ELISA plate reader (iMark^™^ Microplate Absorbance Reader, Bio-RAD, US) by measurement at every hour. Three independent experiments were performed and for each of which, samples were run in duplicates. Growth curves were extracted using Excel (Microsoft, San Diego, CA) along with the exponential equations and coefficients of determination (R^2^) (S1 File).

### *In vivo* experiments

#### Experiment 1: Evaluation of virulence

Seventy-five chickens of a brown egg-producing layer line, susceptible to fowl typhoid and pullorum disease, were used in this study. Birds were obtained on their first day of age from a commercial hatchery where they were vaccinated against Marek’s disease. They were housed in metal cages in a temperature controlled room with a 16-hour light-dark cycle at the School of Agriculture and Veterinary Sciences. On arrival, cloacal swabs were taken and cultured [30] in order to exclude infection by *Salmonella* spp. They received water and antibiotic-free, balanced feed without any nutritional restriction.

At 21 days of age, chickens were randomly distributed in five equal-sized groups and infected as follows: Wild-type SP449/87 (pullorum disease positive control), wild-type SG287/91 (fowl typhoid positive control), *S*. Gallinarum Δ*idnTO* (SGΔ*idnTO*), *S*. Gallinarum Δ*ccmH* (SGΔ*ccmH*) and *S*. Gallinarum double mutant (SGΔ*ccmHidnTO*). One mL aliquots of inocula containing approximately 8 x 10^8^ colony-forming units (CFU) prepared in LB as described previously [4] were inoculated orally into the crop of chickens by oral gavage. Clinical signs were daily recorded for 17 days and those chickens which developed severe clinical signs of fowl typhoid were humanely sacrificed by cervical dislocation. Despite our efforts, some of the chickens used in this study died prior to euthanasia (natural death by fowl typhoid). All dead animals (whether euthanized or not) were necropsied in order to evaluate the presence of gross pathologies.

#### Experiment 2.1: Evaluation of systemic infection

Ninety, one-day-old brown egg-producing layers were obtained from the same commercial establishment and under the same conditions stated above. Birds were housed, checked and reared as detailed above. At 15 days of age, chicks were randomly distributed into the following six equal-sized groups: SP449/87 (pullorum disease positive control), wild-type SG287/91 (fowl typhoid positive control), SGΔ*idnTO*, SGΔ*ccmH*, SGΔ*ccmHidnTO* and non-infected control. Animals were infected as described above. Non-infected animals were inoculated with 1 mL of sterile LB broth. Five chickens of each group were euthanized at 1, 3 and 5 days post-infection (dpi) and samples of spleen and liver were collected for estimation of viable bacteria in tissues. Briefly, tissue samples were macerated with pestle and mortar in PBS (10% w/v) with further10-fold serial dilutions in PBS (v/v). Every dilution was spotted on brilliant green agar (BGA—Oxoid, US) plates, containing 100 μg / mL of nalidixic acid. Plates were allowed to dry followed by incubation at 37 °C for 24 hours. In parallel, the first dilutions were enriched with an equal volume of 2-fold concentrated selenite broth (Oxoid, US) containing 4 μg / mL of novobiocin and incubated at 37 °C for 24 hours. After incubation, the number of CFU per gram of tissue (CFU/g) was estimated. Those samples for which no bacteria grew for counting on BGA were re-streaked onto new BGA plates from the enriched cultures and incubated for 24 hours at 37 °C. If then positive, a value of 10^2^ CFU / g was assigned for the downstream analyses.

#### Experiment 2.2: Evaluation of systemic inflammation and immune response

Caecal tonsils and fragments of spleens were collected at 1, 3 and 5 dpi from three out of five chickens used in the experiment 2.1, flash-frozen in liquid nitrogen and transferred to a freezer at -80 °C until used. For histopathology, fragments of spleen were thawed and fixed in 10% buffered formalin, dehydrated, embedded in paraffin wax, sectioned at 4 μm and stained with haematoxylin and eosin as routinely performed. Blinded sections were analysed by two veterinary pathologists on the microscope and alterations were scored as absent (0), discrete (1), moderate (2) and intense (3). For gene expression analysis, total RNA was purified using the RNeasy Mini Kit (Qiagen, GE) and 0.6 μg was reverse transcribed to cDNA using the QuantiTect Reverse Transcription Kit (Qiagen, GE) following the manufacturer’s instructions. Total RNA was quantified using the DeNovix DS11+ spectrophotometer (DeNovix, US) and qualified by denaturing agarose gel [32]. Reverse transcription quantitative PCR (RT-qPCR) was performed using a RealCFX96 Touch Thermocycler (Bio-Rad, US) with 6.25 μL SYBR Green Jump StartTaqReady Mix (Sigma-Aldrich, US), 0.6 μM of each primer (Sigma-Aldrich, US), about 50 ng template (cDNA) and ultra-pure water (Sigma-Aldrich, US) to a final volume of 12.5 μL. Cycling conditions were 94 °C for 2 min, followed by 40 cycles at 94 °C for 15 s and 58 °C for 30 s. Melting curves were generated after the amplification cycles by gradually increasing the temperature from 65 °C to 95 °C while the signal was taken at each 0.5°C of temperature enhancement.

Selection of suitable reference genes for this study was done by GeNorm [33] and NormFinder [34] software. Transcription stability of glyceraldehyde 3-phosphate dehydrogenase (GAPDH), hypoxanthine-guanine phosphoribosyltransferase (HPRT), β-actin (BACT) and 28S rRNA (28S) were all tested in a representative set of samples and the cycles of quantification (Cq) submitted to analysis. IL-6 and CXCLi2 (previously IL-8) transcription were measured in the harvested caecal tonsils at 1 and 3 dpi and in spleens at 3 dpi, whereas IFNγ transcription were solely assessed in the spleen samples at 5 dpi. The efficiency curves were acquired from a serial dilution of pooled samples comprised of equal volumes of every cDNA, including the negative controls, and new curves were performed for each tested gene every time a new plate was run. Target samples and non-template controls were run in duplicate and efficiency curves in triplicate. Information on the primers used in this step is shown in Table 1.

**Table 1:** Primer sequences for the RT-qPCR analysis performed in this study.

Gene	Sequence (5’->3’)	Amplicon (bp)	Reference
GAPDH	F: GGCACGCCATCACTATC	61	[35]
	R: CCTGCATCTGCCCATTT		
HPRT	F: CCCAAACATTATGCAGACGA	66	[35]
	R: TGTCCTGTCCATGATGAGC		
Β-actin	F: CACAGATCATGTTTGAGACCTT	101	[35]
	R: CATCACAATACCAGTGGTACG		
rRNA 28S	F: GGCGAAGCCAGAGGAAACT	62	[9]
	R: GACGACCGATTTGCACGTC		
CXCLi2	F: GCCCTCCTCCTGGTTTCAG	74	[9]
	R: TGGCACCGCAGCTCATT		
IL6	F: GCTCGCCGGCTTCGA	71	[9]
	R: GGTAGGTCTGAAAGGCGAACAG		
IFNγ	F: GTGAAGAAGGTGAAAGATATCATGGA	71	[9]
	R: GCTTTGCGCTGGATTCTCA		

GAPDH: Glyceraldehyde 3-phosphate dehydrogenase; HPRT: Hypoxanthine-guanine phosphoribosyltransferase; CXCLi2: proinflammatory cytokine previously known as interleukin 8; IL6: Interleukin 6; INFγ: Interferon gamma.

### Statistical analyses

Mortality rates were compared amongst the infected groups by Chi-square test. Statistical differences amongst the CFU recovered from livers and spleens were determined using One-way analysis of variance (ANOVA) followed by Bonferroni correction for multiple comparisons. RT-qPCR data were normalised as previously described by Kogut et al. [36] and subjected to an ANOVA followed by Tukey`s post hoc test analysis for multiple comparisons. Bacterial growth in M9 minimal medium was analysed as follows: the OD_595_ values were plotted by way of Excel software (Microsoft, San Diego, CA) and logarithmic growth phases (log phase) were identified for each culture, re-plotted and fitted to exponential curves. The exponential coefficients were determined and compared by ANOVA followed by Bonferroni correction. Additionally, the OD_595_ were compared by hour of measurement also through ANOVA followed by Bonferroni correction. For histopathology, the average scores of each microscopic alteration were analysed by one-tailed non-parametric Kruskal Wallis test followed by Dunn`s post hoc test analysis for multiple comparisons. Differences were considered to be significant at *P* < 0.05. Statistical analyses were performed and graphs were drawn using the GraphPad Prism 6 for windows, version 6.01.

## Results

### Sequence comparisons

The coding sequences (CDS) of the *idnT* and *idnO* genes were predicted to be 1,221 bp and 765 bp in length, respectively, on the *S*. Gallinarum chromosomes. In *S*. Pullorum, CDS of the *idnT* gene is 607 bp in length due to a 614-bp deletion on its 5 prime-end starting from the start codon, whereas the downstream *idnO* gene is 686 bp because it harbours a 79-bp deletion on the 3 prime-end that removes its stop codon. The *ccmH* gene in turn is duplicated in both biovars so that they were named in this study according to their position in SG287/91 genome (AM933173.1). CDS of the *ccmH*(1) and *ccmH*(2) genes are located between the 3,810,868–3,811,920 and 2,332,348–2,333,400 bases, respectively, and both have 1.053 bp in *S*. Gallinarum. In *S*. Pullorum genomes, however, *ccmH*(1) has 958 bp due to a 95-bp deletion which causes a frameshift that alters the amino acid sequence from the position 418 onwards. This rupture is also present in the *ccmH*(2), which additionally harbours other deletions making it slightly shorter in length. These information are shown as supplementary figures (S2–S5 Files).

### *In vitro* studies

**Fig 1:**
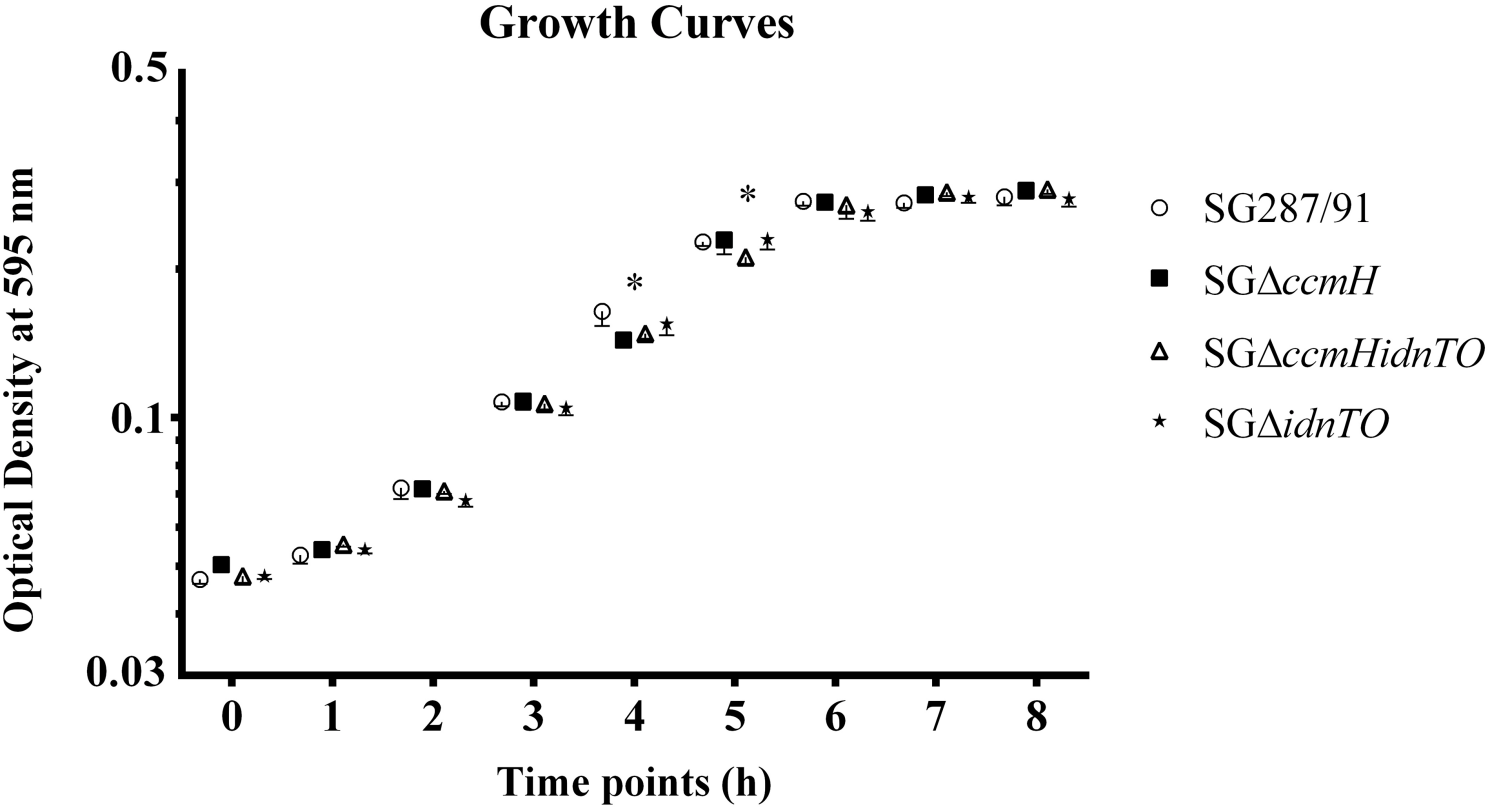
Growth curves of SG287/91 and its derivative mutants cultured in M9 minimal media containing D-gluconate as the sole carbon source. (*) On the comparison by time point, the growth of at least two strains statistically differed by one-way ANOVA followed by Bonferroni correction for multiple comparisons. Symbols on the curve express mean ± standard deviation. SG287/91: *S*. Gallinarum str. 287/91 (parental strain); SGΔ*ccmH*: *S*. Gallinarum str. 287/91 harbouring mutation in the genes *ccmH*; SGΔ*ccmHidnTO*: *S*. Gallinarum str. 287/91 harbouring mutations in the genes *idnTO* and *ccmH*; SGΔ*idnTO*: *S*. Gallinarum str. 287/91 harbouring mutation in the genes *idnTO*.

All tested bacteria were able to grow on BGA at plenty. Analysis on the colony morphology and consistency did not reveal any difference amongst strains. Furthermore, SG287/91 and the genetically engineered strains were able to grow in M9 minimal medium supplemented with D-gluconate as the sole carbon source (Fig 1). Bacteria entered into the logarithmic growth phase at 2 hours of sub-culturing (hsc) and reached the stationary growth phase by 6 hsc. The OD_595_ values from log phase, i.e., the numbers obtained between 2 and 5 hsc, were re-plotted and statistical analysis of the exponential coefficients demonstrated that the tested strains grew at even rates. Comparisons by time point showed that SGΔ*ccmH* and SGΔ*ccmHidnTO* cultures had slightly lower OD_595_ than SG287/91 and SGΔ*idnTO* at 4 hsc. Despite that, only SGΔ*ccmHidnTO* presented lower OD_595_ at 5 hsc. No statistical significance was found for the OD_595_ variations at any other time point.

### *In vivo* experiments

#### Experiment 1: Evaluation of virulence

**Fig 2:**
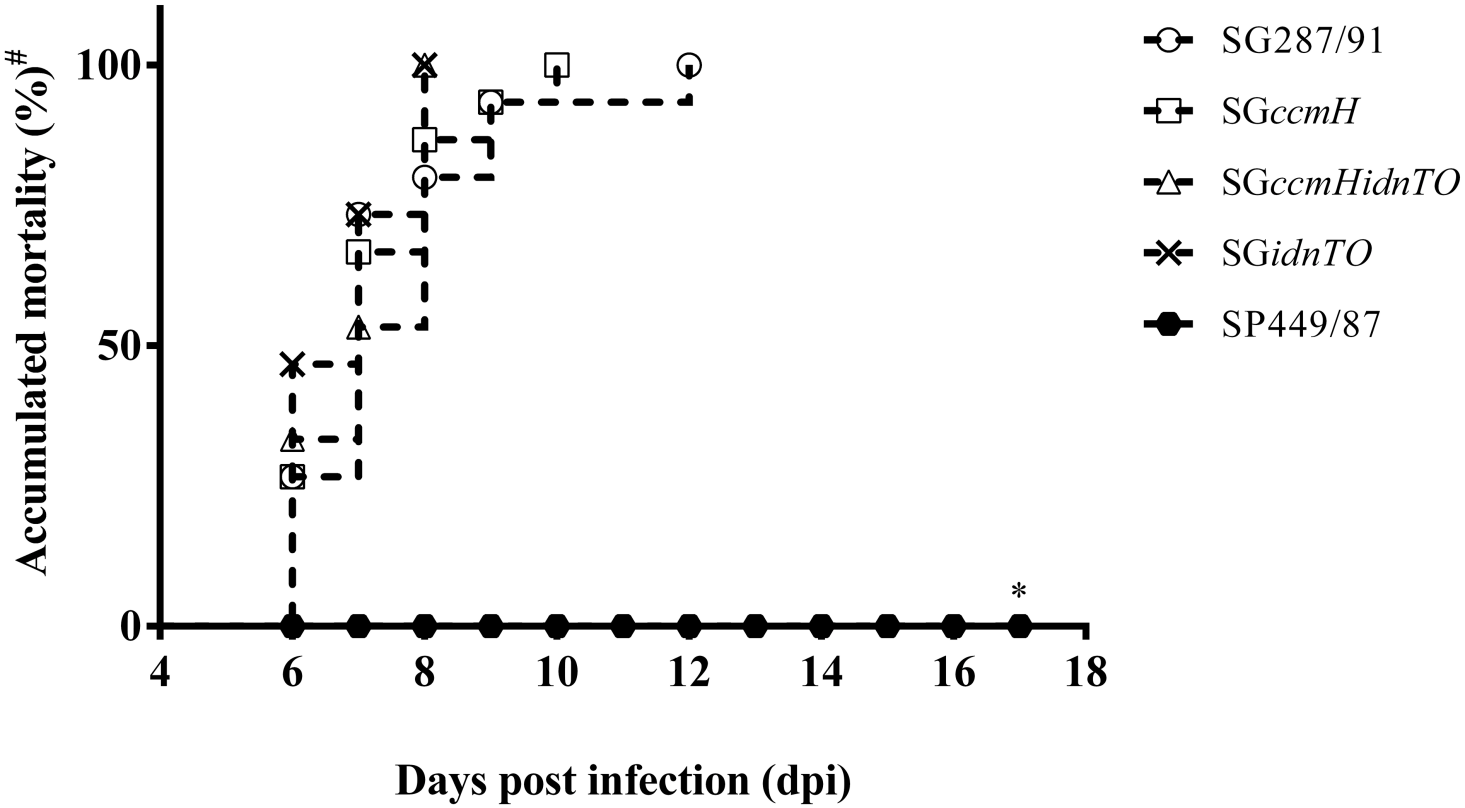
Accumulated deaths throughout the 17-day experiment 1. (**#**) In this study, chickens that became severely ill were euthanized and death counted as a result of fowl typhoid. (*) All *S*. Pullorum-infected stayed alive throughout the experiment so that this group was found to be statistically different from the others. SP449/87: *S*. Pullorum str. 449/87 (pullorum disease positive control); SG287/91: *S*. Gallinarum str. 287/91 (fowl typhoid positive control); SGΔ*ccmH*: *S*. Gallinarum str. 287/91 harbouring mutation in the genes *ccmH*; SGΔ*ccmHidnTO*: *S*. Gallinarum str. 287/91 harbouring both mutations in the genes *idnTO* and *ccmH*; SGΔ*idnTO*: *S*. Gallinarum str. 287/91 harbouring mutation in the genes *idnTO*.

No signs of disease were observed over the first 2 dpi. At 3 dpi, only a few SP449/87-infected animals presented slight clinical signs such as ruffled feathers and depression which completely disappeared from 5 dpi onwards. No mortality was observed amongst the SP449/87-infected chickens over the 17 days of experimentation (Fig 2). In contrast, birds from all *S*. Gallinarum-infected groups began showing clinical signs of fowl typhoid at 4 dpi. Amongst them, green-yellowish diarrhoea, ruffled feathers and anorexia were apparent and became more severe over the following days. Mortality (i.e., either euthanasia of very ill animals or natural deaths) commenced at 6 dpi amongst all *S*. Gallinarum-infected chickens and regardless of the group it reached 100% of the chickens within 12 days post-infection (dpi). Mortality induced by the *idnTO* mutants reached 100% of animals between 6–8 dpi while SG287/91 and SGΔ*ccmH* did so by 12 and 10 dpi, respectively (Fig 1). Despite that, no statistical significance was found when mortality induced by SG287/91 and its derivative engineered strains were compared.

Typical gross pathologies of fowl typhoid such as hepatosplenomegaly, hepatic congestion and diffuse white spots and / or colourless areas on the liver surface were observed to all *S*. Gallinarum-infected birds at necropsy. On the 18^th^ dpi, SP449/87-infected chickens were clinically healthy. They were euthanized and at necropsy, typical white-yellowish nodules in the myocardium and duodenum resembling tumours in addition to hydropericardium were the only gross alterations found in organs (convalescence).

#### Experiment 2.1: Evaluation of systemic infection

**Fig 3:**
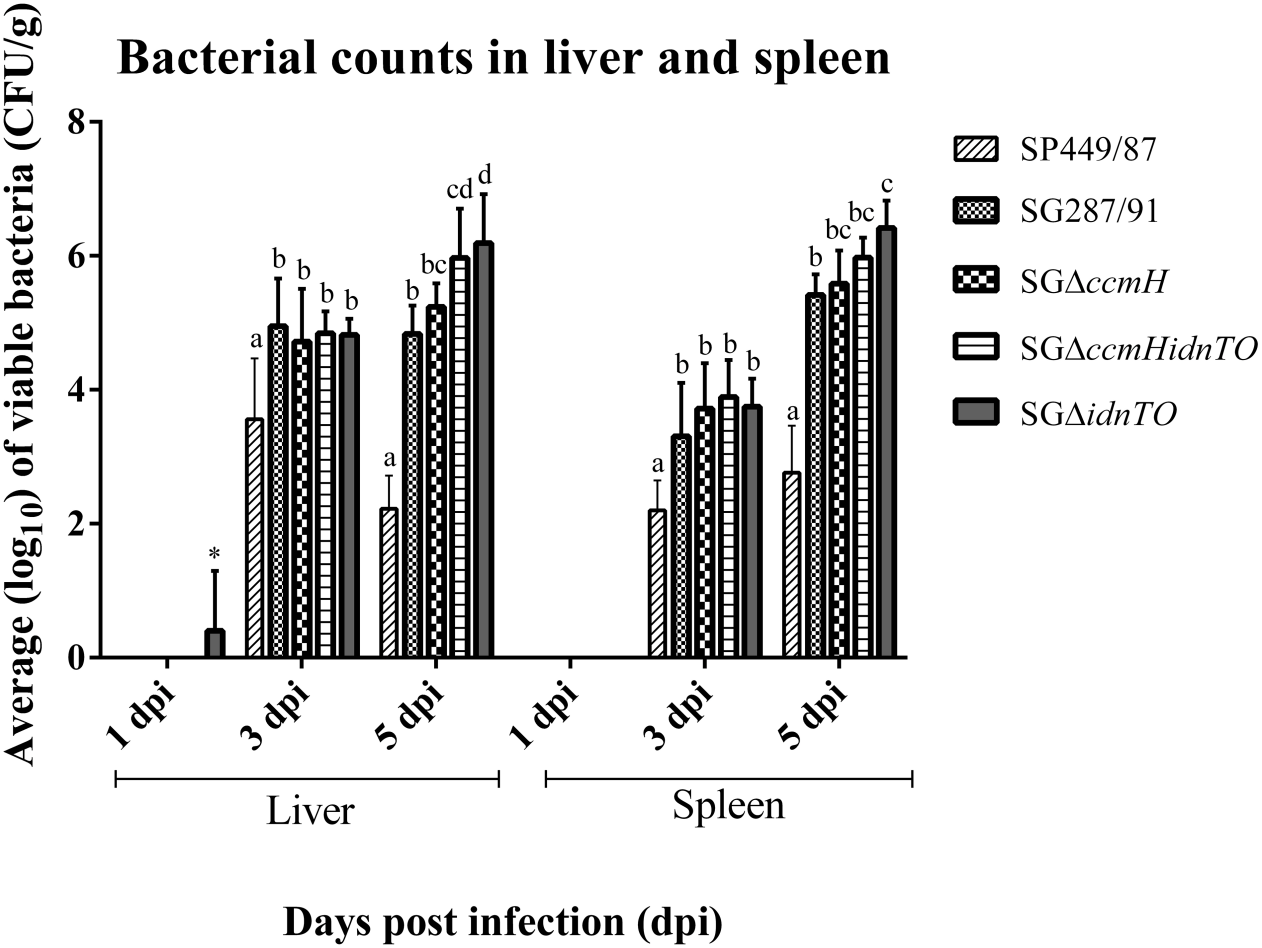
Number of viable colony-forming units (CFU) per gram (g) of liver or spleen tissue collected from chickens infected on the 15 day of age. Organs were collected at 1, 3 and 5 days post-infection (dpi). Graph interpretation: Groups compared by day-post infection for each analysed tissue. Different letters stand for statistically distinct CFU / g through one-way ANOVA followed by Bonferroni correction. Results are expressed as mean ± standard deviation. (*) A single liver sample which was positive for SGΔ*idnTO* after enrichment; SP449/87: *S*. Pullorum str. 449/87 (pullorum disease positive control); SG287/91: *S*. Gallinarum str. 287/91 (fowl typhoid positive control); SGΔ*ccmH*: *S*. Gallinarum str. 287/91 harbouring mutation on the genes *ccmH* alone; SGΔ*idnTO*: *S*. Gallinarum str. 287/91 harbouring mutation on the genes *idnTO* alone; SGΔ*ccmHidnTO*: *S*. Gallinarum str. 287/91 harbouring both mutations.

Fig 3 shows that all bacteria tested in this study were able to invade from the intestine to systemic sites (spleen and liver) over the experiment. No bacteria were detected at 1 dpi in spleen or liver tissues, except for one liver from a SGΔ*idnTO*- infected chicken, which was positive after enrichment. SP449/87 was less invasive than were *S*. Gallinarum strains at both 3 and 5 dpi (*P* < 0.05). At 3 dpi, all tested *S*. Gallinarum strains were recovered at similar quantities from spleens and livers so no statistical difference was found. At 5 dpi, both the *idnTO* mutants were retrieved in higher amounts from livers than SG287/91 while from spleens only SGΔ*idnTO* showed this behaviour in relation to the wild-type strain. These higher recoveries were supported statistically (*P* < 0.05). SGΔ*ccmH* loads did not statistically differ from load of SG287/91 throughout the experiment, in spite of its numerically lower recovery than SGΔ*idnTO* from livers at 5 dpi (*P* < 0.05)

#### Experiment 2.2.1: Evaluation of systemic inflammation

**Fig 4:**
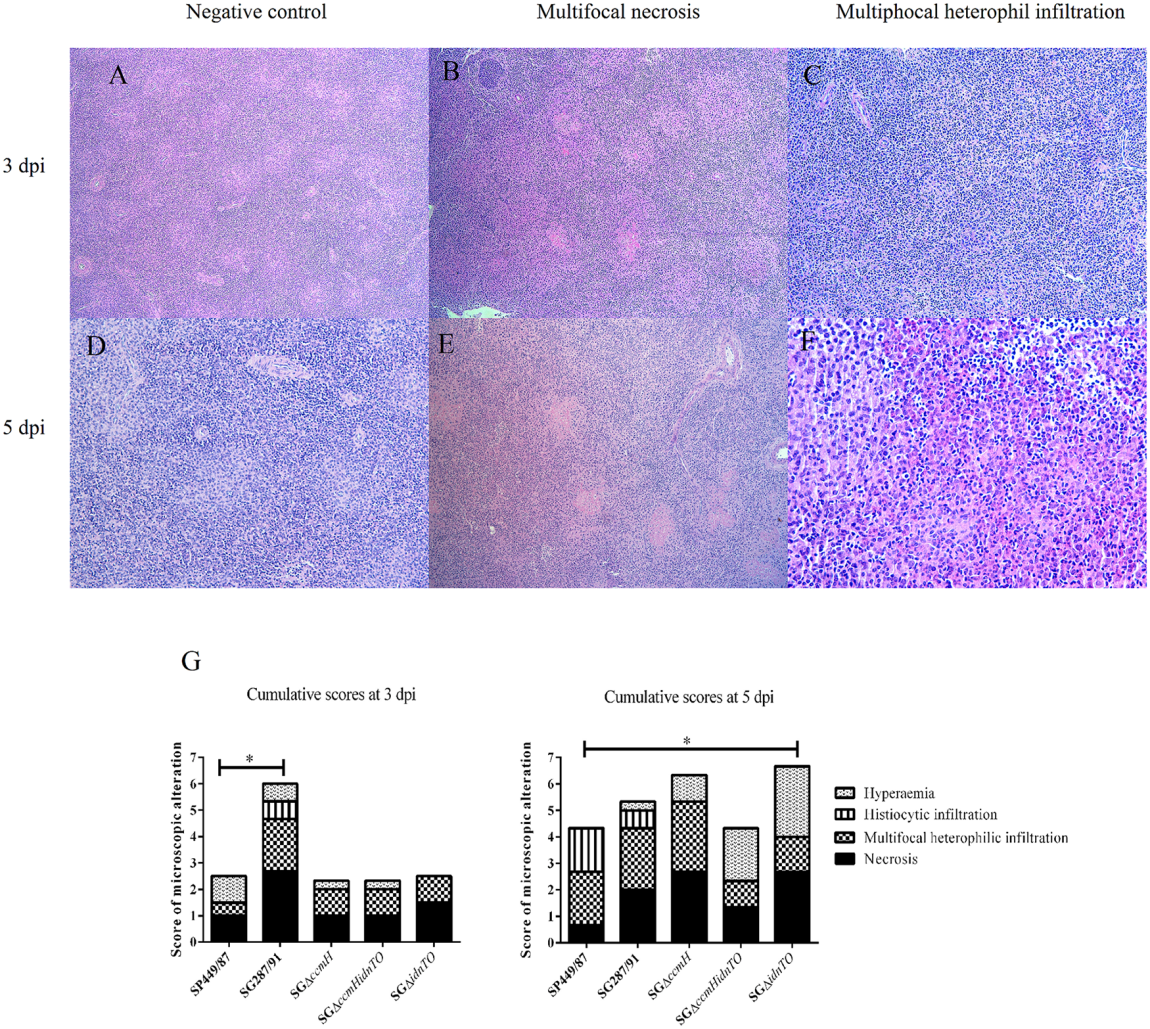
Representative micrographs of the main microscopic alterations found in spleens of the 15-day-old chickens infected in this study. Fig 4A: Spleens of non-infected chickens at 3 dpi (100x magnification); Fig 4B: SG287/91-infected chicken showing moderate to intense multifocal necrosis at 3 dpi (100x magnification); Fig 4C: SG287/91-infected chicken showing moderate to intense multifocal heterophil infiltration at 3 dpi (200x magnification); Fig 4D: Spleens of non-infected chickens at 5 dpi (200x magnification); Fig 4E: SG287/91-infected chicken showing moderate to intense multifocal necrosis at 5 dpi (100x magnification); Fig 4F: SGΔ*ccmH*-infected chicken showing intense multifocal heterophil infiltration at 5 dpi (400x magnification); Fig 4G: Average scores displayed as accumulated graphs. (*) Meaning that at least one of the displayed microscopic alterations statistically differed between the highlighted groups.

Signs of inflammation were observed in the investigated spleen tissues. Multifocal heterophil infiltration and necrosis were the main perceived alterations and their intensity varied depending on the day of infection and on the infecting strain (Fig 4). At 3 dpi, SG287/91 caused intense necrosis (Fig 4B) and moderate multifocal heterophilic infiltration (Fig 4C) in contrast to the other studied bacteria, which provoked only discrete alterations, although statistical significance was solely found for the differences in heterophil infiltration stimulated by SG287/91 (average score: 2.0) and SP449/87 (average score: 0.5) (Fig 4G). At 5 dpi, moderate to intense necrosis was found in spleens of SG287/91-, SGΔ*ccmH*- SGΔ*idnTO*-infected animals (Fig 4E), whereas necrosis triggered by SGΔ*ccmHidnTO* was not very intense and was scored in between those caused by SG287/91 and SP449/87. SGΔ*ccmHidnTO* and SGΔ*idnTO* did not evoke intense heterophil recruitment as did SGΔ*ccmH* (Fig 4F), SG287/91 and SP449/87. Infection triggered by SGΔ*idnTO* led to a higher hyperaemia in spleens at 5 dpi than infection with SP449/87 (*P* < 0.05). Histiocytic infiltrates were observed in a few spleens of SG287/91-infected animals at 3 and 5 dpi whereas both histiocytic and plasmocytic infiltrates were noted in a few spleens of SP449/87-infected chickens at 5 dpi. No significant changes were found in the spleen tissues at 1 dpi.

#### Experiment 2.2.2: Evaluation of immune response

Both reference gene selector software used in this study indicated HPRT as the most stable reference gene amongst those tested. Besides, HRPT and GAPDH were the optimal combination for this study. Thus, they were used to normalise the CXCLi2, IL6 and INFγ gene transcription. Information regarding the primer efficiencies are shown in S3 Table.

**Fig 5:**
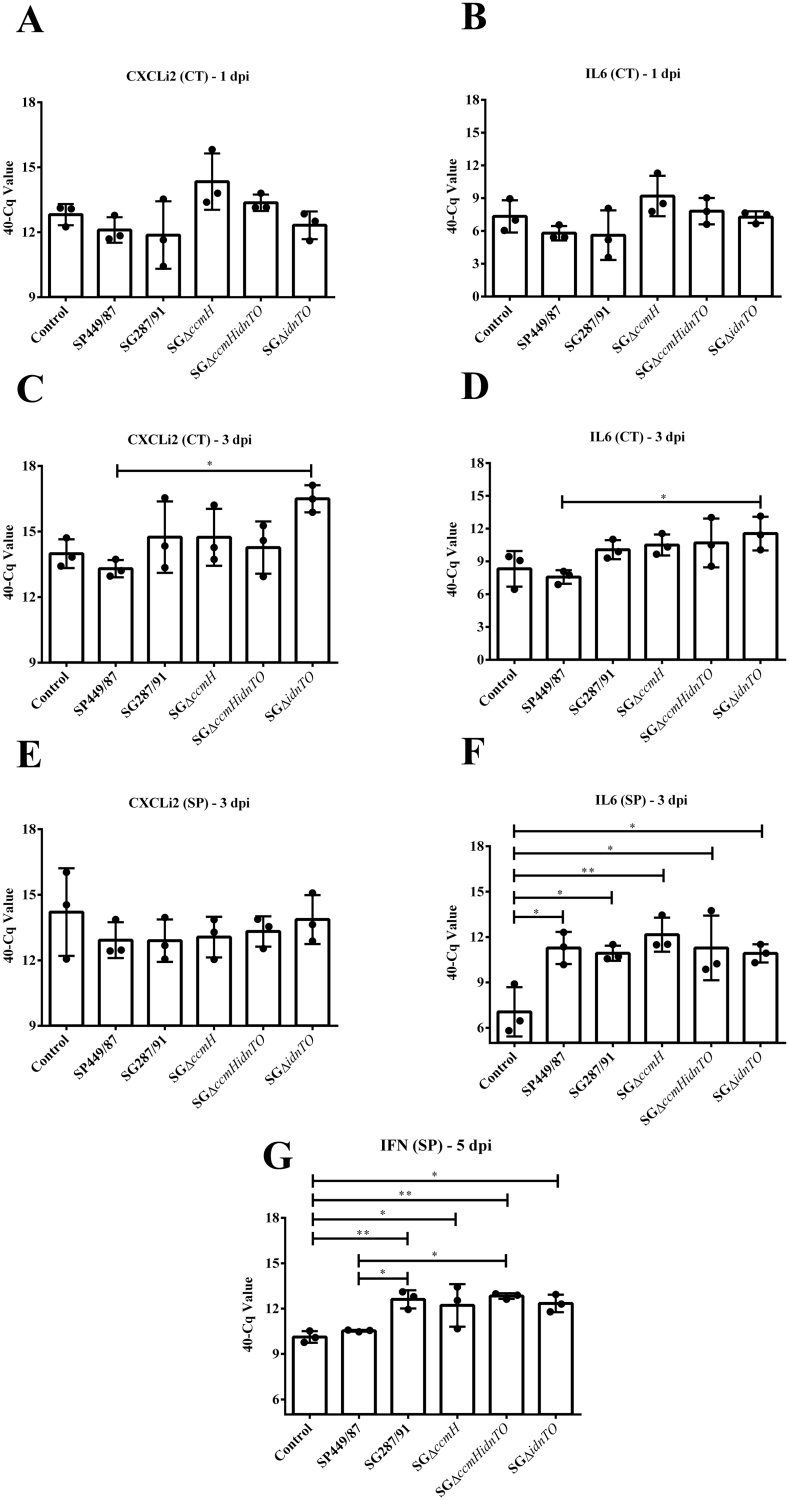
Transcription of the cytokine-related genes evaluated in this study by RT-qPCR. Chickens were orally infected with the strains here tested on the 15 day of age. Samples of caecal tonsils and spleen tissues were collected at 1, 3 and 5 dpi. (*) This symbol shows groups whose mRNA transcription statistically differs by one-way ANOVA followed by Tukey’s test (* *P* < 0.05 / ** *P* < 0.01). CXCLi2: CXC-like chemokine previously named as interleukin 8; IL6: Interleukin 6; IFN: Interferon gamma; (CT): Caecal tonsil; (SP): Spleen; Control: Non-infected chickens; SP449/87: Chickens infected with *S*. Pullorum str. 449/87 (pullorum disease positive control); SG287/91: Chickens infected with *S*. Gallinarum str. 287/91 (fowl typhoid positive control); SGΔ*ccmH*: Chickens infected with SGΔ*ccmH*; SGΔ*idnTO*: Chickens infected with SGΔ*idnTO*; SGΔ*ccmHidnTO*: Chickens infected with SGΔ*ccmHidnTO*.

The pro-inflammatory cytokines measured in this study presented very similar transcription profiles in caecal tonsils (Fig 5A–5D). No statistical significance was found between CXCLi2 and IL6 mRNA levels at 1 dpi. Even so, it is noteworthy that the wild-type strains, SP449/87 and SG287/91, slightly suppressed CXCLi2 and IL6 transcription in comparison with the negative control, while SGΔ*ccmH* and SGΔ*ccmHidnTO* seemed to have stimulated them (Fig 5A and 5B). At 3 dpi, SGΔ*idnTO*-infected chickens transcribed CXCLi2 and IL6 cytokines in caecal tonsils at higher levels than SP449/87-infected animals (*P* < 0.05), but not than non-infected ones (*P* > 0.05) (Fig 5C and 5D). Pair-wise comparisons of CXCLi2 and IL6 transcription amongst SG-infected birds rendered no statistical significance for the differences observed (*P* > 0.05).

In this study, differences in splenic CXCLi2 mRNA quantities did not show statistical significance amongst groups at 3 dpi, though it seemed to be low-transcribed by infected animals (Fig 5E). In contrast, IL6 mRNA was highly transcribed by the infected chickens in comparison with the negative control animals (Fig 5F). In spite of that, no statistical significance was found when groups of infection were compared to each other. At 5 dpi, IFNγ was highly transcribed in the spleens of all *S*. Gallinarum-infected chickens (Fig 5G). In the meantime, SP449/87-infected animals showed IFNγ mRNA levels similar to that observed in the spleens of non-infected birds. In addition, the higher SG287/91- and SGΔ*ccmHidnTO*-induced IFNγ transcription with regard to SP449/87 infection was statistically supported (Fig 5G). Noteworthy, SGΔ*idnTO* also induced IFNγ transcription which nearly differed from that induced by SP449/87 (*P* = 0.065).

## Discussion

Fowl typhoid caused by *Salmonella* Gallinarum is an infection of poultry that concerns the poultry industry in developing countries, since it has been considered under control in the developed ones [1,5]. Contrary to the agent of pullorum disease, *S*. Pullorum, which establishes a more intimate and evolved interaction with the host, *S*. Gallinarum rapidly leads to a severe systemic disease that may result in high mortality depending on the infecting strain and host genetic background [2]. To date, the genetic mechanisms for such different patterns of pathogenicity are obscure. Thus, in the present study we evaluated the contribution of the candidate genes *idnT* and *idnO* (*idnTO*), and *ccmH* to *S*. Gallinarum pathogenicity.

These target genes were previously highlighted as conserved pseudogenes in *S*. Pullorum genomes that would have encoded proteins involved in bacterial metabolism [16]. They are part of a core set comprised of 469 coding sequences (CDS) that undergo degradation in the genomes of host-adapted *Salmonella enterica* subsp. *enterica* serovars, named by Nuccio and Bäumler [14] as extraintestinal pathovars. *S*. Gallinarum and *S*. Pullorum are both members of this group, though the *idnTO* and *ccmH* genes have suffered no decay on *S*. Gallinarum chromosomes.

The findings of the present study seem to suggest that the alteration introduced in the *idnTO* and *ccmH* genes would have little effect on the *S*. Gallinarum pathogenicity. In spite of harbouring genetic alterations, colonies grew as expected on agar plates, showed the same rate of growth in D-glucose- (data not shown) or D-gluconate-supplemented M9 minimal medium, caused similar mortality in susceptible chickens and triggered almost identical patterns of immune response. Despite this overall perspective, it is noteworthy that SGΔ*idnTO* and SGΔ*ccmHidnTO* showed altered interaction with susceptible chickens orally infected as they were capable of multiplying in liver or spleen tissues at a higher rate than did SG287/91 at 5 dpi. Moreover, in comparison with the wild-type strain (SG287/91), SGΔ*idnTO* and SGΔ*ccmHidnTO* elicited microscopic alterations of different magnitudes such as a lighter multifocal heterophilic infiltration at 5 dpi; a day before the onset of mortality when various animals had already developed typical and severe clinical signs of fowl typhoid. Altogether, these data indicate that the host-pathogen interaction triggered by the *idnTO* mutants slightly changed, though with no significant impact on the course of fowl typhoid according to the model used in this study. It is interesting though that contrary to our expectations the *idnTO* mutants have increased their virulence to susceptible birds during late infection, suggesting that the metabolic restriction imposed by mutation induced greater expression of virulence factors. The methods here applied and the obtained results, however, do not provide a rationale for the fitness improvement observed. The *idnTO* genes were predicted to be related to L-idonate uptake in an *E*. *coli* model [28]; a substrate that was not tested in the present study. Therefore, future studies will be needed to elucidate this phenomenon.

Discrepancies between *in vitro* and *in vivo* results were also reported by Jelsbak et al. [21] that tested the importance of the *purN* and *purT* genes, which participate in the purine biosynthesis pathway, to *S*. Typhimurium str. 4/74 infection in mice. The PurN and PurT enzymes possessed redundant functions *in vitro* but not *in vivo*. According to these authors, these inconsistencies may arise from the fact that some functional auxotrophy may appear when metabolites are present at levels below an important threshold during infection of the host [21]. This is of special interest in the present study because SGΔ*idnTO* and SGΔ*ccmHidnTO* were not supposed to cease the uptake of D-gluconate nor its metabolism as the *gnt* genes, which play roles in the usage of such a carbohydrate through the main system (GntI), are intact in their genomes (data not shown). As a matter of fact, no differences in the overall bacterial growth in M9 medium plus D-gluconate were noticed. Even though, SGΔ*idnTO* and SGΔ*ccmHidnTO* infections presented some particularities, indicating that the restriction imposed through gene disruption induced differential expression of virulence factors whether through D-gluconate limitations (levels below the threshold *in vivo*) or by other yet unknown mechanism.

On the other hand, mutations introduced to the *ccmH* genes showed ambiguous effect on the SGΔ*ccmH* pathogenicity in the susceptible birds. Mortality and bacterial counts at systemic sites (liver and spleen) were similar between SGΔ*ccmH* and SG287/91 infections. There was a tendency between the *ccmH* clones to increase CXCLi2 and IL6 transcription in caecal tonsils at 1 dpi whereas SP449/87 and SG287/91 suppressed them. As already mentioned, the *ccmH* genes seem to be required during anaerobiosis [36] a condition largely found in the intestines [37]. Thus, any expected effect of this mutation should appear by the time bacteria were crossing the gut barriers rather than when they had already reached the liver and spleen tissues. Despite this, the increased CXCLi2 and IL6 transcription in caecal tonsils was neither statistically supported nor showed any impacted on fowl typhoid pathogenesis indicating that not even an auxiliary function might be attributed to *ccmH* genes for *S*. Gallinarum pathogenicity.

It was postulated by Nuccio and Bäumler [14] that some of the pseudogenes in the extraintestinal pathovars would mirror genes important to their ancestor to survive in the host gastrointestinal tract but that are not useful as they occupy the systemic niche as parasites of mononuclear phagocytic cells. There are a few known pseudogenes in *S*. Gallinarum/Pullorum genomes that are related to intestinal survival. As an example, the *ttr* operon, which encodes enzymes to provide an alternative electron acceptor allowing *Salmonella* spp. to outgrow the commensal organism in the inflamed intestine [38], harbours inactivating mutations in *S*. Gallinarum str. 287/91 chromosome [13]. Thus, it is possible that *ccmH* and *idnTO* genes are relics from the ancestor genome with no practical function in fowl typhoid pathogenesis. Curiously, Li et al. [11] theorized that *S*. Pullorum lineage has gone through a rapid rate of evolution so it adapted to birds earlier than *S*. Gallinarum; such a genetic process hints at the reason whereby the *ccmH* and *idnTO* genes have already been under degradation in the former lineage and that they may also be in *S*. Gallinarum genomes in the future. Furthermore, reconstruction of these genes on *S*. Pullorum chromosome would show whether bacterium loses fitness, raising a reason for their negative selection in this biovar.

Finally, infection of birds with SG287/91 and its derivative mutants elicited similar immune responses. IFNγ is a differential marker between *S*. Gallinarum and *S*. Pullorum infections and was highly transcribed at 5 dpi in spleens of all *S*. Gallinarum-infected chicken but not by SP449/87-infected animals. It suggests that a more complex mechanism than inactivation of a few genes might be driving the distinct host-pathogen interaction of these bacteria with birds. Despite this, the possibility that some of the previously identified biovar-specific pseudogenes [16] could play a part in this process cannot be completely discarded since only two of them were tested in the present study. Thus, further investigations on this gene core could increase our knowledge of fowl typhoid pathogenesis and assist on the comprehension of the distinct host-pathogen relationship triggered by *S*. Gallinarum and *S*. Pullorum in susceptible birds.

## Supporting information

S1 Table Primers used in this study. (DOCX)

S2 Table Details on the mutations introduced to the *itnTO* and *ccmH* genes. (DOCX)

S3 Table Information obtained from the efficiency (E) curves which were further used to normalise the RT-qPCR data from this study. (DOCX)

S1 File Exponential curves, equations and coefficients of determination (R2) extracted from the bacterial growth. (PDF)

S2 File Alignment of *idnT* CDS from *S*. Gallinarum strains 287/91 (SG287_91) and 9184 (SG9184), and *S*. Pullorum strains CDC1983-67 (SPCDC) and RKS5078 (SPRKS). (PDF)

S3 File Alignment of *idnO* CDS from *S*. Gallinarum strains 287/91 (SG287_91) and 9184 (SG9184), and *S*. Pullorum strains CDC1983-67 (SPCDC) and RKS5078 (SPRKS). (PDF)

S4 File Alignment of *ccmH*(1) CDS from *S*. Gallinarum strains 287/91 (SG287_91) and 9184 (SG9184), and *S*. Pullorum strains CDC1983-67 (SPCDC) and RKS5078 (SPRKS). (PDF)

S5 File Alignment of *ccmH*(2) CDS from *S*. Gallinarum strains 287/91 (SG287_91) and 9184 (SG9184), and *S*. Pullorum strains CDC1983-67 (SPCDC) and RKS5078 (SPRKS). (PDF)
