# Supplementary material for: Evaluation of pathogenicity of Salmonella Gallinarum strains harbouring deletions in genes whose orthologues are conserved pseudogenes in S. Pullorum
Source: PLoS One. 2018 Jul 20;13(7):e0200585. doi: 10.1371/journal.pone.0200585 (PMC6054384; doi:10.1371/journal.pone.0200585)
Supplement: S1 Table — (DOCX) [file pone.0200585.s001.docx]

S1 Table. Primers used in this study.

| **Primer identification^a^** | **Sequence (5’→3’)^b^** | **Position^c^** | **Source** |
| --- | --- | --- | --- |
| idnT(50)-F | GTTTCTCTCGTCAAAAGCCTCCGACTTTGTTAACGGACATCTGCTGTTTG**GTGTAGGCTGGAGCTGCTTC** | 4540181..4540132 | This study |
| idnT(50)-R | ATGGCCATCAGAATGACCGGGATCACGGCGGCAAAAATACTGTTCCAGAA**CATATGAATATCCTCCTTAG** | 4539329..4539378 | This study |
| ccmH(50)-F | CGGGTGGATAATCGTTGCCCGCACGCGCCGGCGGGTGCGCCTGCGCCGGG**GTGTAGGCTGGAGCTGCTTC** | 2333035..2332986 / 3811233..3811282 | This study |
| ccmH(50)-R | CGGATACTGCGCTCTATCACCGCCCGCCGGGCGTCACCCGCCGGCAGCAGT**CATATGAATATCCTCCTTAG** | 2332367..2332417 / 3811901..3811851 | This study |
| idnTO(test)-F | ACGGAGATGACCAAAGCGCT | 4540291..4540272 | This study |
| idnTO(test)-R | TATATTGGCCCACGCCGCTA | 4539069..4539088 | This study |
| ccmH(test1B)-R | ATCATACTGCACACGGGCTT | 3812011..3811992 | This study |
| ccmH(test1B)-F | GTACTATGAGGCGGAGGTGC | 3810487..3810506 | This study |
| ccmH(test2B)-F | ATAGCGATCCGGAAGTGCTG | 2331985..2332004 | This study |
| ccmH(test2B)-R | CTACGGCAACTTCGTCACCT | 2333131..2333112 | This study |
| K1 | CAGTCATAGCCGAATAGCCT | – | [26] |
| K2 | CGGTGCCCTGAATGAACTGC | – | [26] |
| C1 | TTATACGCAAGGCGACAAGG | – | [26] |
| C2 | GATCTTCCGTCACAGGTAGG | – | [26] |

F: Forward primer; R: Reverse primer; K1 and K2: checking kanamycin-based mutation(s); C1 and C2: checking chloramphenicol-based mutation(s).

^a^ gene(50): hybrid primers used to construct mutants by lambda Red-mediated recombination; gene(test): primers used to check the constructs.

^b^ The twenty base pairs highlighted in bold are utilised to amplify the chloramphenicol or kanamycin gene sequences from the pKD3 or pKD4 plasmid, respectively.

^c^ Positions mapped on *S*. Gallinarum str. 287/91 chromosome (GenBank: AM933173.1)
