# Supplementary material for: Evaluation of pathogenicity of Salmonella Gallinarum strains harbouring deletions in genes whose orthologues are conserved pseudogenes in S. Pullorum
Source: PLoS One. 2018 Jul 20;13(7):e0200585. doi: 10.1371/journal.pone.0200585 (PMC6054384; doi:10.1371/journal.pone.0200585)
Supplement: S2 Table — (DOCX) [file pone.0200585.s002.docx]

S2 Table. Details on the mutations introduced to the *itnTO* and *ccmH* genes.

| **Gene^a^** | **Mutation position^b^** | **Deletion length** | **Observations** |
| --- | --- | --- | --- |
| *idnTO* | 4540131..4539379 | 753 bp | Small deletion on the 3’ end of *idnO* and large deletion from the 5’ end of *idnT* genes. The intergenic region was also deleted. |
| *ccmH*(1) | 3811283..3811850 | 568 bp | Large deletion within the *ccmH* genes. *ccmH*(2) is located upstream of the *nap* operon whereas *ccmH*(1) is located downstream of the *tor* operon. |
| *ccmH*(2) | 2332985..2332418 | 568 bp |  |

^a^ *ccm*: cytochrome c maturation; *idn*: L-idonate metabolism;

^b^ Positions mapped on *S*. Gallinarum str. 287/91 chromosome (GenBank: AM933173.1);

bp: base pairs.
