# Supplementary material for: Evaluation of pathogenicity of Salmonella Gallinarum strains harbouring deletions in genes whose orthologues are conserved pseudogenes in S. Pullorum
Source: PLoS One. 2018 Jul 20;13(7):e0200585. doi: 10.1371/journal.pone.0200585 (PMC6054384; doi:10.1371/journal.pone.0200585)
Supplement: S3 Table — (DOCX) [file pone.0200585.s003.docx]

S3 Table. Information obtained from the efficiency (E) curves which were further used to normalise the RT-qPCR data from this study.

| **Gene** | **Organ** | **Efficiency (E)** | **Coefficient of correlation (R^2^)** |
| --- | --- | --- | --- |
| HPRT | Caecal tonsil (CT) | 98,80% | 0,991 |
|  | Spleen (SP) | 94,88% | 0,986 |
| GAPDH | Caecal tonsil (CT) | 96,72% | 0,941 |
|  | Spleen (SP) | 104,39% | 0,987 |
| CXCLi2 | Caecal tonsil (CT) | 102,13% | 0,991 |
|  | Spleen (SP) | 100,33% | 0,995 |
| IL6 | Caecal tonsil (CT) | 103,27% | 0,989 |
|  | Spleen (SP) | 101,14% | 0,983 |
| β-actin | –^a^ | 94,66% | 0,998 |
| r RNA 28S | –^a^ | 64,20% | 0,998 |
| IFNγ | Spleen (SP) | 99,54% | 0,983 |

GAPDH: Glyceraldehyde 3-phosphate dehydrogenase; HPRT: Hypoxanthine-guanine phosphoribosyltransferase; CXCLi2: proinflammatory cytokine previously known as interleukin 8; IL6: Interleukin 6; INFγ: Interferon gamma.

^a^ Data on the transcription of these genes were utilised in the reference gene selection step only, since they were not selected as between the most stables.
