# Supplementary material for: Evaluation of pathogenicity of Salmonella Gallinarum strains harbouring deletions in genes whose orthologues are conserved pseudogenes in S. Pullorum
Source: PLoS One. 2018 Jul 20;13(7):e0200585. doi: 10.1371/journal.pone.0200585 (PMC6054384; doi:10.1371/journal.pone.0200585)

S1 File. Exponential curves, equations and coefficients of determination (R2) extracted from the bacterial growth in D-gluconate

Time	SG287/91-1	SG287/91-2	SG287/91-3
2	0,076	0,069	0,071
3	0,109	0,109	0,105
4	0,168	0,173	0,152
5	0,225	0,232	0,224

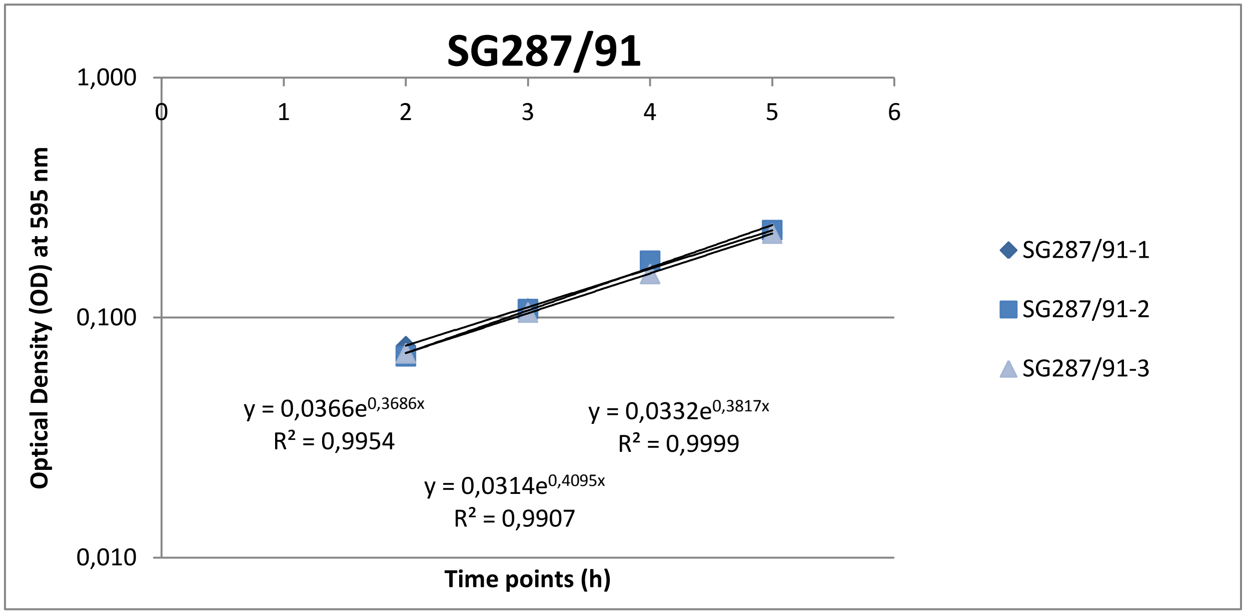

Time	SGΔccmH-1	SGΔccmH-2	SGΔccmH-3
2	0,071	0,073	0,071
3	0,111	0,110	0,103
4	0,145	0,142	0,144
5	0,242	0,232	0,213

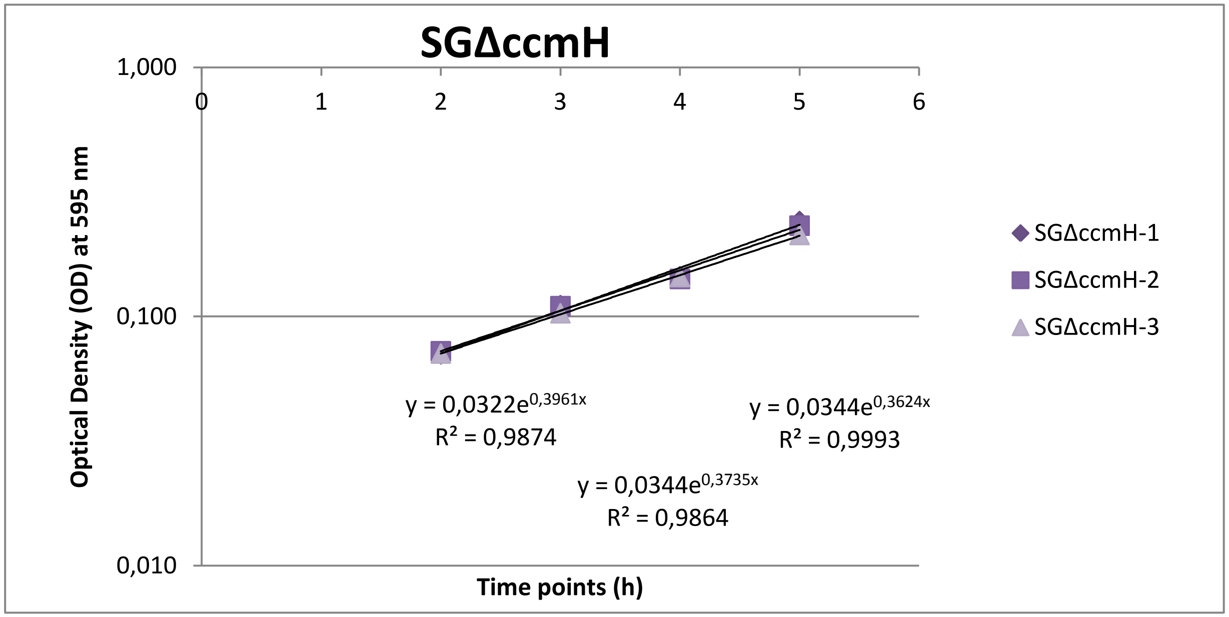

Time	SGΔccmHidnTO-1	SGΔccmHidnTO-2	SGΔccmHidnTO-3
2	0,071	0,070	0,072
3	0,110	0,105	0,105
4	0,152	0,147	0,145
5	0,217	0,203	0,214

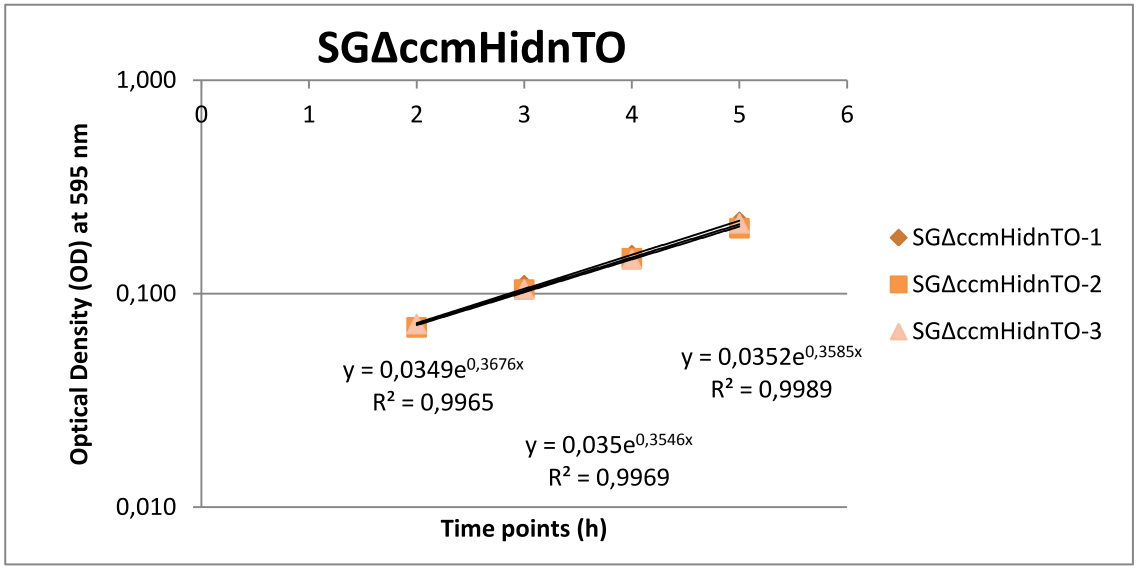

Time	SGΔidnTO-1	SGΔidnTO-2	SGΔidnTO-3
2	0,068	0,066	0,070
3	0,108	0,105	0,101
4	0,157	0,162	0,146
5	0,233	0,238	0,218

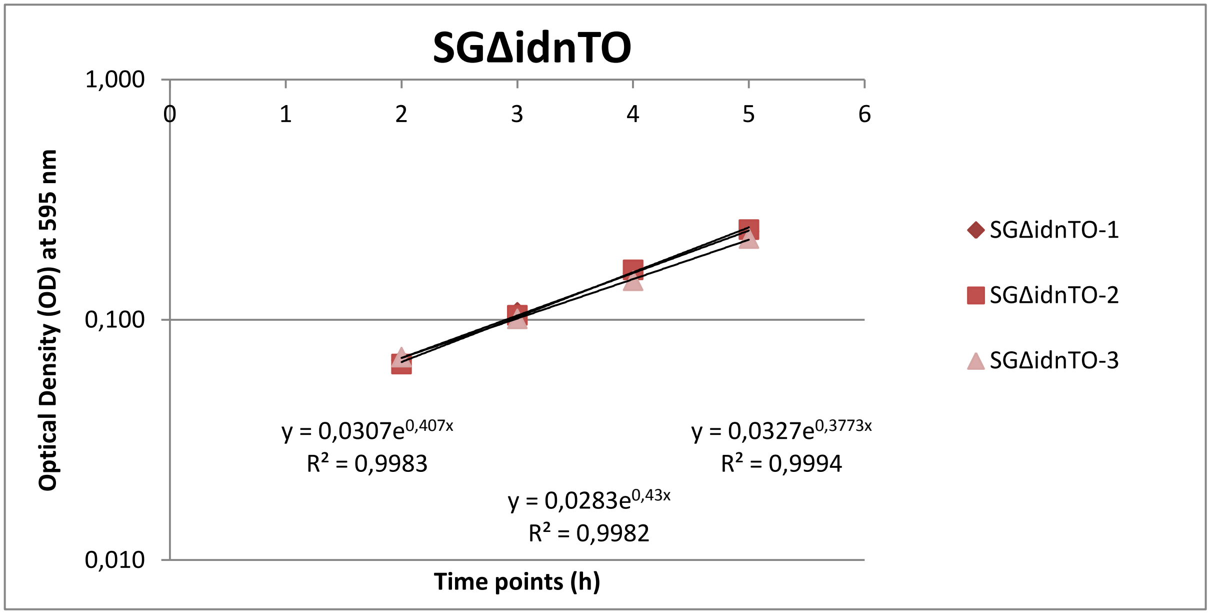

Supplement: S1 File — (PDF) [file pone.0200585.s004.pdf]
