# Supplementary material for: Evaluation of pathogenicity of Salmonella Gallinarum strains harbouring deletions in genes whose orthologues are conserved pseudogenes in S. Pullorum
Source: PLoS One. 2018 Jul 20;13(7):e0200585. doi: 10.1371/journal.pone.0200585 (PMC6054384; doi:10.1371/journal.pone.0200585)
Supplement: S2 File — (PDF) [file pone.0200585.s005.pdf]

S2 File. Alignment of *idnT* CDS from *S*. Gallinarum strains 287/91 (SG287_91) and 9184 (SG9184), and *S*. Pullorum strains CDC1983-67 (SPCDC) and RKS5078 (SPRKS).

```
                                       20                  40
SG287_91_idnT  GTGGTGGGATTTGCCGAAGGGATGGGCGCGCAAGACGTCCTGCACTCCATACAAAACGG 59
  SG9184_idnT  GTGGTGGGATTTGCCGAAGGGATGGGCGCGCAAGACGTCCTGCACTCCATACAAAACGG 59
SPCDC_idnT-RC  -----------------------------------------------------------
SPRKS-idnT-RC  -----------------------------------------------------------

               60                  80                  100
SG287_91_idnT  AATTGGCGGTACTCTGGGCGGACTCGCCATGATCCTCGGTTTTGGCGCGATGCTGGGAA 118
  SG9184_idnT  AATTGGCGGTACTCTGGGCGGACTCGCCATGATCCTCGGTTTTGGCGCGATGCTGGGAA 118
SPCDC_idnT-RC  -----------------------------------------------------------
SPRKS-idnT-RC  -----------------------------------------------------------

                120                 140                 160
SG287_91_idnT  GATTAATTTCCGATACCGGCGCCGCGCAGCGCATTGCGACGACGCTCATTAATACGTTC 177
  SG9184_idnT  GATTAATTTCCGATACCGGCGCCGCGCAGCGCATTGCGACGACGCTCATTAATACGTTC 177
SPCDC_idnT-RC  -----------------------------------------------------------
SPRKS-idnT-RC  -----------------------------------------------------------

                 180                 200                 220
SG287_91_idnT  GGCAAAAAACGCGTGCAATGGGCGCTGGTGATCACCGGGCTTATCGTCGGCCTGGCAAT 236
  SG9184_idnT  GGCAAAAAACGCGTGCAATGGGCGCTGGTGATCACCGGGCTTATCGTCGGCCTGGCAAT 236
SPCDC_idnT-RC  -----------------------------------------------------------
SPRKS-idnT-RC  -----------------------------------------------------------

                  240                 260                 280
SG287_91_idnT  GTTTTTTGAAGTCGGATTCGTACTGCTGTTGCCGCTCGTTTTTACGATTGTGGCCTCAT 295
  SG9184_idnT  GTTTTTTGAAGTCGGATTCGTACTGCTGTTGCCGCTCGTTTTTACGATTGTGGCCTCAT 295
SPCDC_idnT-RC  -----------------------------------------------------------
SPRKS-idnT-RC  -----------------------------------------------------------

                   300                 320                 340
SG287_91_idnT  CGGGACTGCCCTTGCTGTATGTCGGGGTTCCAATGGTCGCCGCCTTATCCGTTACGCAC 354
  SG9184_idnT  CGGGACTGCCCTTGCTGTATGTCGGGGTTCCAATGGTCGCCGCCTTATCCGTTACGCAC 354
SPCDC_idnT-RC  -----------------------------------------------------------
SPRKS-idnT-RC  -----------------------------------------------------------

                    360                 380                 400
SG287_91_idnT  TGCTTCCTGCCGCCGCATCCCGGCCCTACCGCTATTGCGACAATTTTTGAGGCAAATCT 413
  SG9184_idnT  TGCTTCCTGCCGCCGCATCCCGGCCCTACCGCTATTGCGACAATTTTTGAGGCAAATCT 413
SPCDC_idnT-RC  -----------------------------------------------------------
SPRKS-idnT-RC  -----------------------------------------------------------

                     420                 440                 460
SG287_91_idnT  CGGTACGACCCTGCTGTATGGGTTAATCATTACCATACCGACCGTGATTGTTGCCGGCC 472
  SG9184_idnT  CGGTACGACCCTGCTGTATGGGTTAATCATTACCATACCGACCGTGATTGTTGCCGGCC 472
SPCDC_idnT-RC  -----------------------------------------------------------
SPRKS-idnT-RC  -----------------------------------------------------------

                      480                 500                 520
SG287_91_idnT  CGCTGTTTTCAAAATTGCTGGCGCGCTTTGAGAAAGCGCCGCCTGAAGGATTATTTAAT 531
  SG9184_idnT  CGCTGTTTTCAAAATTGCTGGCGCGCTTTGAGAAAGCGCCGCCTGAAGGATTATTTAAT 531
SPCDC_idnT-RC  -----------------------------------------------------------
SPRKS-idnT-RC  -----------------------------------------------------------

                       540                 560                 580
SG287_91_idnT  CCTCATCTATTCAGTGAAGAAGAGATGCCGTCATTCTGGAACAGTATTTTTGCCGCCGT 590
  SG9184_idnT  CCTCATCTATTCAGTGAAGAAGAGATGCCGTCATTCTGGAACAGTATTTTTGCCGCCGT 590
SPCDC_idnT-RC  -----------------------------------------------------------
SPRKS-idnT-RC  -----------------------------------------------------------
```

```
                600                 620                 640
SG287_91_idnT  GATCCCGGTCATTCTGATGGCCATCGCAGCGGTATGTGAAATCACGCTGCCAAAAACGA 649
SG9184_idnT    GATCCCGGTCATTCTGATGGCCATCGCAGCGGTATGTGAAATCACGCTGCCAAAAACGA 649
SPCDC_idnT-RC  ------------------------CGCAGCGGTATGTGAAATCACGCTGCCAAAAACGA 35
SPRKS-idnT-RC  ------------------------CGCAGCGGTATGTGAAATCACGCTGCCAAAAACGA 35
                 660                 680                 700
SG287_91_idnT  ATGCCGTGCGGGTTTTCTTCGAATTTATCGGTAATCCAGCAGTGGCGTTGTTTATTGCC 708
SG9184_idnT    ATGCCGTGCGGGTTTTCTTCGAATTTATCGGTAATCCAGCAGTGGCGTTGTTTATTGCC 708
SPCDC_idnT-RC  ATGCCGTGCGGGTTTTCTTCGAATTTATCGGTAATCCAGCAGTGGCGTTGTTTATTGCC 94
SPRKS-idnT-RC  ATGCCGTGCGGGTTTTCTTCGAATTTATCGGTAATCCAGCAGTGGCGTTGTTTATTGCC 94
                  720                 740                 760
SG287_91_idnT  ATTATCATCGCCATTTTTACGCTGGGCCGACGCAACGGACGTACGGTTGAGCAGGTGAT 767
SG9184_idnT    ATTATCATCGCCATTTTTACGCTGGGCCGACGCAACGGACGTACGGTTGAGCAGGTGAT 767
SPCDC_idnT-RC  ATTATCATCGCCATTTTTACGCTGGGCCGACGCAACGGACGTACGGTTGAGCAGGTGAT 153
SPRKS-idnT-RC  ATTATCATCGCCATTTTTACGCTGGGCCGACGCAACGGACGTACGGTTGAGCAGGTGAT 153
                   780                 800                 820
SG287_91_idnT  GGATATCGTCGGCGAGTCCATTGGCGCTATTGCGATGATTGTTTTTATCATCGCGGGCG 826
SG9184_idnT    GGATATCGTCGGCGAGTCCATTGGCGCTATTGCGATGATTGTTTTTATCATCGCGGGCG 826
SPCDC_idnT-RC  GGATATCGTCGGCGAGTCCATTGGCGCTATTGCGATGATTGTTTTTATCATCGCGGGCG 212
SPRKS-idnT-RC  GGATATCGTCGGCGAGTCCATTGGCGCTATTGCGATGATTGTTTTTATCATCGCGGGCG 212
                    840                 860                 880
SG287_91_idnT  GCGGCGCGTTTAAACAAGTGCTGGTGGATAGCGGCGTGGGCCAATATATCTCGCAATTA 885
SG9184_idnT    GCGGCGCGTTTAAACAAGTGCTGGTGGATAGCGGCGTGGGCCAATATATCTCGCAATTA 885
SPCDC_idnT-RC  GCGGCGCGTTTAAACAAGTGCTGGTGGATAGCGGCGTGGGCCAATATATCTCGCAATTA 271
SPRKS-idnT-RC  GCGGCGCGTTTAAACAAGTGCTGGTGGATAGCGGCGTGGGCCAATATATCTCGCAATTA 271
                     900                 920                 940
SG287_91_idnT  ATGACCGGCACCTCGCTATCTCCGTTATTAATGTGCTGGACGGTAGCCGCCGTGCTGCG 944
SG9184_idnT    ATGACCGGCACCTCGCTATCTCCGTTATTAATGTGCTGGACGGTAGCCGCCGTGCTGCG 944
SPCDC_idnT-RC  ATGACCGGCACCTCGCTATCTCCGTTATTAATGTGCTGGACGGTAGCCGCCGTGCTGCG 330
SPRKS-idnT-RC  ATGACCGGCACCTCGCTATCTCCGTTATTAATGTGCTGGACGGTAGCCGCCGTGCTGCG 330
                      960                 980                 1.000
SG287_91_idnT  TATTGCGTTAGGTTCCGCGACCGTGGCCGCGATAACCACAGCGGGCGTCGTATTGCCGA 1003
SG9184_idnT    TATTGCGTTAGGTTCCGCGACCGTGGCCGCGATAACCACAGCGGGCGTCGTATTGCCGA 1003
SPCDC_idnT-RC  TATTGCGTTAGGTTCCGCGACCGTGGCCGCGATAACCACAGCGGGCGTCGTATTGCCGA 389
SPRKS-idnT-RC  TATTGCGTTAGGTTCCGCGACCGTGGCCGCGATAACCACAGCGGGCGTCGTATTGCCGA 389
                       1.020               1.040               1.060
SG287_91_idnT  TTATTAACGTAACCCACGCCGATCCGGCGTTAATGGTACTGGCGACAGGCGCGGGAAGC 1062
SG9184_idnT    TTATTAACGTAACCCACGCCGATCCGGCGTTAATGGTACTGGCGACAGGCGCGGGAAGC 1062
SPCDC_idnT-RC  TTATTAACGTAACCCACGCCGATCCGGCGTTAATGGTACTGGCGACAGGCGCGGGAAGC 448
SPRKS-idnT-RC  TTATTAACGTAACCCACGCCGATCCGGCGTTAATGGTACTGGCGACAGGCGCGGGAAGC 448
                        1.080               1.100               1.120
SG287_91_idnT  GTTATTGCATCACATGTTAACGATCCCGGTTTTTGGCTGTTTAAAGGGTATTTTAATCT 1121
SG9184_idnT    GTTATTGCATCACATGTTAACGATCCCGGTTTTTGGCTGTTTAAAGGGTATTTTAATCT 1121
SPCDC_idnT-RC  GTTATTGCATCACATGTTAACGATCCCGGTTTTTGGCTGTTTAAAGGGTATTTTAATCT 507
SPRKS-idnT-RC  GTTATTGCATCACATGTTAACGATCCCGGTTTTTGGCTGTTTAAAGGGTATTTTAATCT 507
                         1.140               1.160               1.180
SG287_91_idnT  TAGCGTCGGGGAAACCCTACGTACCTGGACCGTTATGGAAACATTGATTTCTGTCATGG 1180
SG9184_idnT    TAGCGTCGGGGAAACCCTACGTACCTGGACCGTTATGGAAACATTGATTTCTGTCATGG 1180
SPCDC_idnT-RC  TAGCGTCGGGGAAACCCTACGTACCTGGACCGTTATGGAAACATTGATTTCTGTCATGG 566
SPRKS-idnT-RC  TAGCGTCGGGGAAACCCTACGTACCTGGACCGTTATGGAAACATTGATTTCTGTCATGG 566
                          1.200               1.220
SG287_91_idnT  GGTTACTGGGCGTACTGGCGCTGAATGCGGTTCTGCATTAA 1221
SG9184_idnT    GGTTACTGGGCGTACTGGCGCTGAATGCGGTTCTGCATTAA 1221
SPCDC_idnT-RC  GGTTACTGGGCGTACTGGCGCTGAATGCGGTTCTGCATTAA 607
SPRKS-idnT-RC  GGTTACTGGGCGTACTGGCGCTGAATGCGGTTCTGCATTAA 607
```
