# Supplementary material for: Evaluation of pathogenicity of Salmonella Gallinarum strains harbouring deletions in genes whose orthologues are conserved pseudogenes in S. Pullorum
Source: PLoS One. 2018 Jul 20;13(7):e0200585. doi: 10.1371/journal.pone.0200585 (PMC6054384; doi:10.1371/journal.pone.0200585)
Supplement: S3 File — (PDF) [file pone.0200585.s006.pdf]

S3 File. Alignment of *idnO* CDS from *S*. Gallinarum strains 287/91 (SG287_91) and 9184 (SG9184), and *S*. Pullorum strains CDC1983-67 (SPCDC) and RKS5078 (SPRKS).

```
                             20                                      40
SG287_91_idnO   ATGAACGATCTTTTTTCACTGGCAGGTAAAAATATCCTTATCACCGGAGCCGCTCAGGG 59
SG9184_idnO     ATGAACGATCTTTTTTCACTGGCAGGTAAAAATATCCTTATCACCGGAGCCGCTCAGGG 59
SPCDC_idnO-RC   ATGAACGATCTTTTTTCACTGGCAGGTAAAAATATCCTTATCACCGGAGCCGCTCCGGG 59
SPRKS_idnO-RC   ATGAACGATCTTTTTTCACTGGCAGGTAAAAATATCCTTATCACCGGAGCCGCTCCGGG 59
                60                   80                  100
SG287_91_idnO   AATTGGTTATTTGCTGGCAACCGGTCTTGGTCGCTATGGCGCGCGCATTATTGTTAACG 118
SG9184_idnO     AATTGGTTATTTGCTGGCAACCGGTCTTGGTCGCTATGGCGCGCGCATTATTGTTAACG 118
SPCDC_idnO-RC   AATTGGTTATTTGCTGGCAACCGGTCTTGGTCGCTATGGCGCGCGCATTATTGTTAACG 118
SPRKS_idnO-RC   AATTGGTTATTTGCTGGCAACCGGTCTTGGTCGCTATGGCGCGCGCATTATTGTTAACG 118
                  120                 140                 160
SG287_91_idnO   ATATTACTCCGGAGCGCGCCGAAACAGCCGTGACGAAACTTCAGCAGGAAGGGATAAAG 177
SG9184_idnO     ATATTACTCCGGAGCGCGCCGAAACAGCCGTGACGAAACTTCAGCAGGAAGGGATAAAG 177
SPCDC_idnO-RC   ATATTACTCCGGAGCGCGCCGAAACAGCCGTGACGAAACTTCAGCAGGAAGGGATAAAG 177
SPRKS_idnO-RC   ATATTACTCCGGAGCGCGCCGAAACAGCCGTGACGAAACTTCAGCAGGAAGGGATAAAG 177
                    180                 200                 220
SG287_91_idnO   GCTATTGCCGCTCCCTTTAATGTCACCCATAAACAGGATATTGAAGCTGCGATTGAACA 236
SG9184_idnO     GCTATTGCCGCTCCCTTTAATGTCACCCATAAACAGGATATTGAAGCTGCGATTGAACA 236
SPCDC_idnO-RC   GCTATTGCCGCTCCCTTTAATGTCACCCATAAACAGGATATTGAAGCTGCGATTGAACA 236
SPRKS_idnO-RC   GCTATTGCCGCTCCCTTTAATGTCACCCATAAACAGGATATTGAAGCTGCGATTGAACA 236
                      240                 260                 280
SG287_91_idnO   TATCGAAAAAGATATCGGCGTCATTGACGTCCTGATAAATAACGCCGGTATCCAGCGCC 295
SG9184_idnO     TATCGAAAAAGATATCGGCGTCATTGACGTCCTGATAAATAACGCCGGTATCCAGCGCC 295
SPCDC_idnO-RC   TATCGAAAAAGATATCGGCGTCATTGACGTCCTGATAAATAACGCCGGTATCCAGCGCC 295
SPRKS_idnO-RC   TATCGAAAAAGATATCGGCGTCATTGACGTCCTGATAAATAACGCCGGTATCCAGCGCC 295
                        300                 320                 340
SG287_91_idnO   GTCATCCGTTTACTGAGTTTCCCGAGCAGGAGTGGAATGATGTCATCGCCGTAAATCAG 354
SG9184_idnO     GTCATCCGTTTACTGAGTTTCCCGAGCAGGAGTGGAATGATGTCATCGCCGTAAATCAG 354
SPCDC_idnO-RC   GTCATCCGTTTACTGAGTTTCCCGAGCAGGAGTGGAATGATGTCATCGCCGTAAATCAG 354
SPRKS_idnO-RC   GTCATCCGTTTACTGAGTTTCCCGAGCAGGAGTGGAATGATGTCATCGCCGTAAATCAG 354
                          360                 380                 400
SG287_91_idnO   ACCGCGGTTTTTCTCGTCTCTCAGGCCGTTACGCGCCGTATGGTGGCGCGTAAGGCAGG 413
SG9184_idnO     ACCGCGGTTTTTCTCGTCTCTCAGGCCGTTACGCGCCGTATGGTGGCGCGTAAGGCAGG 413
SPCDC_idnO-RC   ACCGCGGTTTTTCTCGTCTCTCAGGCCGTTACGCGCCGTATGGTGGCGCGTAAGGCAGG 413
SPRKS_idnO-RC   ACCGCGGTTTTTCTCGTCTCTCAGGCCGTTACGCGCCGTATGGTGGCGCGTAAGGCAGG 413
                            420                 440                 460
SG287_91_idnO   AAAAGTGATCAATATCTGTTCGATGCAAAGCGAGCTGGGCCGCGACACGATTACGCCAT 472
SG9184_idnO     AAAAGTGATCAATATCTGTTCGATGCAAAGCGAGCTGGGCCGCGACACGATTACGCCAT 472
SPCDC_idnO-RC   AAAAGTGATCAATATCTGTTCGATGCAAAGCGAGCTGGGCCGCGACACGATTACGCCAT 472
SPRKS_idnO-RC   AAAAGTGATCAATATCTGTTCGATGCAAAGCGAGCTGGGCCGCGACACGATTACGCCAT 472
                              480                 500                 520
SG287_91_idnO   ATGCCGCGTCGAAGGGTGCCGTGAAGATGCTGACTCGCGGCATGTGCGTGGAGCTGGCG 531
SG9184_idnO     ATGCCGCGTCGAAGGGTGCCGTGAAGATGCTGACTCGCGGCATGTGCGTGGAGCTGGCG 531
SPCDC_idnO-RC   ATGCCGCGTCGAAGGGTGCCGTGAAGATGCTGACTCGCGGCATGTGCGTGGAGCTGGCG 531
SPRKS_idnO-RC   ATGCCGCGTCGAAGGGTGCCGTGAAGATGCTGACTCGCGGCATGTGCGTGGAGCTGGCG 531
                                540                 560                 580
SG287_91_idnO   CGTCATAATATCCAGGTCAACGGTATTGCGCCGGGATACTTCAAAACGGAGATGACCAA 590
SG9184_idnO     CGTCATAATATCCAGGTCAACGGTATTGCGCCGGGATACTTCAAAACGGAGATGACCAA 590
SPCDC_idnO-RC   CGTCATAATATCCAGGTCAACGGTATTGCGCCGGGATACTTCAAAACGGAGATGACCAA 590
SPRKS_idnO-RC   CGTCATAATATCCAGGTCAACGGTATTGCGCCGGGATACTTCAAAACGGAGATGACCAA 590
```

```
                                   600                                   620                                   640
                                    |                                     |                                     |
SG287_91_idnO    AGCGCTGGTTGAAGATGAAGCCTTCACCTCCTGGCTATGCAAACGTACGCCTGCCGCAC 649
SG9184_idnO      AGCGCTGGTTGAAGATGAAGCCTTCACCTCCTGGCTATGCAAACGTACGCCTGCCGCAC 649
SPCDC_idnO-RC    AGCGCTGGTTGAAGATGAAGCCTTCACCTCCTGGCTATGCAAACGTACGCCTGCCGCAC 649
SPRKS_idnO-RC    AGCGCTGGTTGAAGATGAAGCCTTCACCTCCTGGCTATGCAAACGTACGCCTGCCGCAC 649

                                     660                                   680                                   700
                                      |                                     |                                     |
SG287_91_idnO    GCTGGGGCGATCCCCAGGAGCTTATTGGCGCAGCGGTGTTTCTCTCGTCAAAAGCCTCC 708
SG9184_idnO      GCTGGGGCGATCCCCAGGAGCTTATTGGCGCAGCGGTGTTTCTCTCGTCAAAAGCCTCC 708
SPCDC_idnO-RC    GCTGGGGCGATCCCCGGGAGCTTATTGGCGCAGCGGT---------------------- 686
SPRKS_idnO-RC    GCTGGGGCGATCCCCGGGAGCTTATTGGCGCAGCGGT---------------------- 686

                                       720                                   740                                   760
                                        |                                     |                                     |
SG287_91_idnO    GACTTTGTTAACGGACATCTGCTGTTTGTCGATGGCGGTATGCTGGTTGCCGTCTGA 765
SG9184_idnO      GACTTTGTTAACGGACATCTGCTGTTTGTCGATGGCGGTATGCTGGTTGCCGTCTGA 765
SPCDC_idnO-RC    --------------------------------------------------------- 686
SPRKS_idnO-RC    --------------------------------------------------------- 686
```
