# Supplementary material for: Evaluation of pathogenicity of Salmonella Gallinarum strains harbouring deletions in genes whose orthologues are conserved pseudogenes in S. Pullorum
Source: PLoS One. 2018 Jul 20;13(7):e0200585. doi: 10.1371/journal.pone.0200585 (PMC6054384; doi:10.1371/journal.pone.0200585)
Supplement: S4 File — (PDF) [file pone.0200585.s007.pdf]

S4 File. Alignment of *ccmH*(1) CDS from *S*. Gallinarum strains 287/91 (SG287_91) and 9184 (SG9184), and *S*. Pullorum strains CDC1983-67 (SPCDC) and RKS5078 (SPRKS).

```
                                        20                                        40
SG287_91_ccmH(1)  ATGAGACTGTTACCGGGCATGGTGATGCTGATGCTGGTGCTGGTTATCTCCGGGTCA 57
  SG9184_ccmH(1)  ATGAGACTGTTACCGGGCATGGTGATGCTGATGCTGGTGCTGGTTATCTCCGGGTCA 57
   SPCDC_ccmH(1)  ATGAGACTGTTACCGGGCATGGTGATGCTGATGCTGGTGCTGGTTATCTCCGGGTCA 57
   SPRKS_ccmH(1)  ATGAGACTGTTACCGGGCATGGTGATGCTGATGCTGGTGCTGGTTATCTCCGGGTCA 57
                      60                                        80                                        100
SG287_91_ccmH(1)  GCGCGGGCGACCACCGACGTGATGCCGTTTAAAGATGAAGCGCAGGAGCAGCAGTTC 114
  SG9184_ccmH(1)  GCGCGGGCGACCACCGACGTGATGCCGTTTAAAGATGAAGCGCAGGAGCAGCAGTTC 114
   SPCDC_ccmH(1)  GCGCGGGCGACCACCGACGTGATGCCGTTTAAAGATGAAGCGCAGGAGCAGCAGTTC 114
   SPRKS_ccmH(1)  GCGCGGGCGACCACCGACGTGATGCCGTTTAAAGATGAAGCGCAGGAGCAGCAGTTC 114
                         120                                       140                                       160
SG287_91_ccmH(1)  CGCCAGCTCACGGAGCAGCTGCGCTGCCCGAAATGCCAGAACAACAGCATTGCGGAC 171
  SG9184_ccmH(1)  CGCCAGCTCACGGAGCAGCTGCGCTGCCCGAAATGCCAGAACAACAGCATTGCGGAC 171
   SPCDC_ccmH(1)  CGCCAGCTCACGGAGCAGCTGCGCTGCCCGAAATGCCAGAACAACAGCATTGCGGAC 171
   SPRKS_ccmH(1)  CGCCAGCTCACGGAGCAGCTGCGCTGCCCGAAATGCCAGAACAACAGCATTGCGGAC 171
                            180                                       200                                       220
SG287_91_ccmH(1)  TCGAACGCGATGATAGCCACCGACATGCGCCGCAGGGTGTATGACCTGATGCAGGAG 228
  SG9184_ccmH(1)  TCGAACGCGATGATAGCCACCGACATGCGCCGCAGGGTGTATGACCTGATGCAGGAG 228
   SPCDC_ccmH(1)  TCGAACGCGATGATAGCCACCGACATGCGCCGCAGGGTGTATGACCTGATGCAGGAG 228
   SPRKS_ccmH(1)  TCGAACGCGATGATAGCCACCGACATGCGCCGCAGGGTGTATGACCTGATGCAGGAG 228
                               240                                       260                                       280
SG287_91_ccmH(1)  GGGAAGAGCCGCCAGGAAATCATCGATTACATGGTGGCGCGCTACGGCAACTTCGTC 285
  SG9184_ccmH(1)  GGGAAGAGCCGCCAGGAAATCATCGATTACATGGTGGCGCGCTACGGCAACTTCGTC 285
   SPCDC_ccmH(1)  GGGAAGAGCCGCCAGGAAATCATCGATTACATGGTGGCGCGCTACGGCAACTTCGTC 285
   SPRKS_ccmH(1)  GGGAAGAGCCGCCAGGAAATCATCGATTACATGGTGGCGCGCTACGGCAACTTCGTC 285
                                  300                                       320                                       340
SG287_91_ccmH(1)  ACCTACGACCCGCCGCTGACCCCGCTGACCCCGCTGACGGTGCTGCTGTGGGTGCTG 342
  SG9184_ccmH(1)  ACCTACGACCCGCCGCTGACCCCGCTGACCCCGCTGACGGTGCTGCTGTGGGTGCTG 342
   SPCDC_ccmH(1)  ACCTACGACCCGCCGCTGACCCCGCTGACCCCGCTGACGGTGCTGCTGTGGGTGCTG 342
   SPRKS_ccmH(1)  ACCTACGACCCGCCGCTGACCCCGCTGACCCCGCTGACGGTGCTGCTGTGGGTGCTG 342
                                     360                                       380
SG287_91_ccmH(1)  CCGCTGGCCGCCATCGTGGCGGGCGGGTGGATAATCGTTGCCCGCACGCGCCGGCGG 399
  SG9184_ccmH(1)  CCGCTGGCCGCCATCGTGGCGGGCGGGTGGATAATCGTTGCCCGCACGCGCCGGCGG 399
   SPCDC_ccmH(1)  CCGCTGGCCGCTATCGTGGCGGGCGGGTGGATAATCGTTGCCCGCACGCGCCGGCGG 399
   SPRKS_ccmH(1)  CCGCTGGCCGCTATCGTGGCGGGCGGGTGGATAATCGTTGCCCGCACGCGCCGGCGG 399
                  400                                       420                                       440
SG287_91_ccmH(1)  GTGCGCCTGCGCCGGGAGCCGCTGCCGGCGGACACCCCGGTTTGCGGCGCGCGCGCC 456
  SG9184_ccmH(1)  GTGCGCCTGCGCCGGGAGCCGCTGCCGGCGGACACCCCGGTTTGCGGCGCGCGCGCC 456
   SPCDC_ccmH(1)  GTGCGCCTGCGCCGG------------------------------------------ 414
   SPRKS_ccmH(1)  GTGCGCCTGCGCCGG------------------------------------------ 414
                     460                                       480                                       500
SG287_91_ccmH(1)  GGGTGGGGCGTTTACGTGCCGGGGGCCGTCATTGCGCTGGCGGTCGGCGCCGGCAGC 513
  SG9184_ccmH(1)  GGGTGGGGCGTTTACGTGCCGGGGGCCGTCATTGCGCTGGCGGTCGGCGCCGGCAGC 513
   SPCDC_ccmH(1)  -----------------------------------------------------CAGC 418
   SPRKS_ccmH(1)  -----------------------------------------------------CAGC 418
                        520                                       540                                       560
SG287_91_ccmH(1)  TACGCCCTGACCGGCAGCTATCAGCAGGTCAGGGCCTGGCAGCAGGCAACGGCGCAG 570
  SG9184_ccmH(1)  TACGCCCTGACCGGCAGCTATCAGCAGGTCAGGGCCTGGCAGCAGGCAACGGCGCAG 570
   SPCDC_ccmH(1)  TACGCCCTGACCGGCAGCTATCAGCAGGTCAGGGCCTGGCAGCAGGCAACGGCGCAG 475
   SPRKS_ccmH(1)  TACGCCCTGACCGGCAGCTATCAGCAGGTCAGGGCCTGGCAGCAGGCAACGGCGCAG 475
```

```
                                         580                                      600                                      620
                                          |                                        |                                        |
SG287_91_ccmH(1)  ACGCCCGGGCTGCTGGCGCGGGCGCTGGACCCGGCGGCGCAGCCGCTGAATGAAGAG 627
  SG9184_ccmH(1)  ACGCCCGGGCTGCTGGCGCGGGCGCTGGACCCGGCGGCGCAGCCGCTGAATGAAGAG 627
   SPCDC_ccmH(1)  ACGCCCGGGCTGCTGGCGCGGGCGCTGGACCCGGCGGCGCAGCCGCTGAATGAAGAG 532
   SPRKS_ccmH(1)  ACGCCCGGGCTGCTGGCGCGGGCGCTGGACCCGGCGGCGCAGCCGCTGAATGAAGAG 532
                                            640                                      660                                      680
                                             |                                        |                                        |
SG287_91_ccmH(1)  GAGATGGCGCGGCTGGCGCTGGGGCTGCGCACCCGCCTGCAGAATGATGCCGGCAAT 684
  SG9184_ccmH(1)  GAGATGGCGCGGCTGGCGCTGGGGCTGCGCACCCGCCTGCAGAATGATGCCGGCAAT 684
   SPCDC_ccmH(1)  GAGATGGCGCGGCTGGCGCTGGGGCTGCGCACCCGCCTGCAGAATGATGCCGGCAAT 589
   SPRKS_ccmH(1)  GAGATGGCGCGGCTGGCGCTGGGGCTGCGCACCCGCCTGCAGAATGATGCCGGCAAT 589
                                               700                                      720                                      740
                                                |                                        |                                        |
SG287_91_ccmH(1)  GTTGAGGGCTGGCTCATGCTGGGGCGCACCGGTATGGTACTGGGTAATGCCGGTACC 741
  SG9184_ccmH(1)  GTTGAGGGCTGGCTCATGCTGGGGCGCACCGGTATGGTACTGGGTAATGCCGGTACC 741
   SPCDC_ccmH(1)  GTTGAGGGCTGGCTCATGCTGGGGCGCACCGGTATGGTACTGGGTAATGCCGGTACC 646
   SPRKS_ccmH(1)  GTTGAGGGCTGGCTCATGCTGGGGCGCACCGGTATGGTACTGGGTAATGCCGGTACC 646
                                                  760                                      780
                                                   |                                        |
SG287_91_ccmH(1)  GCCACCGGGGCCTATGCGAACGCCTACCGCCTGGACCCGAAAAACAGCGATGCGGCG 798
  SG9184_ccmH(1)  GCCACCGGGGCCTATGCGAACGCCTACCGCCTGGACCCGAAAAACAGCGATGCGGCG 798
   SPCDC_ccmH(1)  GCCACCGGGGCCTATGCGAACGCCTACCGCCTGGACCCGAAAAACAGCGATGCGGCG 703
   SPRKS_ccmH(1)  GCCACCGGGGCCTATGCGAACGCCTACCGCCTGGACCCGAAAAACAGCGATGCGGCG 703
                     800                                      820                                      840
                      |                                        |                                        |
SG287_91_ccmH(1)  CTGGGCTACGCGGAGGCGCTGACGCGCTCGTCCGACCCGGAGGATAACCGCGGCGGC 855
  SG9184_ccmH(1)  CTGGGCTACGCGGAGGCGCTGACGCGCTCGTCCGACCCGGAGGATAACCGCGGCGGC 855
   SPCDC_ccmH(1)  CTGGGCTACGCGGAGGCGCTGACGCGCTCGTCCGACCCGGAGGATAACCGCCGCGGC 760
   SPRKS_ccmH(1)  CTGGGCTACGCGGAGGCGCTGACGCGCTCGTCCGACCCGGAGGATAACCGCCGCGGC 760
                        860                                      880                                      900
                         |                                        |                                        |
SG287_91_ccmH(1)  GGGGAGCTGCTGCGTCAGCTGGTGAGAAGTGACCACACGGATATCCGGGTGTTAAGC 912
  SG9184_ccmH(1)  GGGGAGCTGCTGCGTCAGCTGGTGAGAAGTGACCACACGGATATCCGGGTGTTAAGC 912
   SPCDC_ccmH(1)  GGGGAGCTGCTGCGTCAGCTGGTGAGAAGTGACCACACGGATATCCGGGTGTTAAGC 817
   SPRKS_ccmH(1)  GGGGAGCTGCTGCGTCAGCTGGTGAGAAGTGACCACACGGATATCCGGGTGTTAAGC 817
                           920                                      940                                      960
                            |                                        |                                        |
SG287_91_ccmH(1)  CTGTATGCGTTCAGCGCCTTTGAGCAGCAGCGTTTTGGCGAGGCGGTGGCGGCCTGG 969
  SG9184_ccmH(1)  CTGTATGCGTTCAGCGCCTTTGAGCAGCAGCGTTTTGGCGAGGCGGTGGCGGCCTGG 969
   SPCDC_ccmH(1)  CTGTATGCGTTCAGCGCCTTTGAGCAGCAGCGTTTTGGCGAGGCGGTGGCGGCCTGG 874
   SPRKS_ccmH(1)  CTGTATGCGTTCAGCGCCTTTGAGCAGCAGCGTTTTGGCGAGGCGGTGGCGGCCTGG 874
                              980                                    1.000                                    1.020
                               |                                        |                                        |
SG287_91_ccmH(1)  GAGATGATGCTGAAACTGCTGCCGGCGGGTGACGCCCGGCGGGCGGTGATAGAGCGC 1026
  SG9184_ccmH(1)  GAGATGATGCTGAAACTGCTGCCGGCGGGTGACGCCCGGCGGGCGGTGATAGAGCGC 1026
   SPCDC_ccmH(1)  GAGATGATGCTGAAACTGCTGCCGGCGGGTGACGCCCGGCGGGCGGTGATAGAGCGC 931
   SPRKS_ccmH(1)  GAGATGATGCTGAAACTGCTGCCGGCGGGTGACGCCCGGCGGGCGGTGATAGAGCGC 931
                                1.040
                                  |
SG287_91_ccmH(1)  AGTATCCGGCTGGCGCAGGAGAAATAA 1053
  SG9184_ccmH(1)  AGTATCCGGCTGGCGCAGGAGAAATAA 1053
   SPCDC_ccmH(1)  AGTATCCGGCTGGCGCAGGAGAAATAA 958
   SPRKS_ccmH(1)  AGTATCCGGCTGGCGCAGGAGAAATAA 958
```
