# Supplementary material for: Evaluation of pathogenicity of Salmonella Gallinarum strains harbouring deletions in genes whose orthologues are conserved pseudogenes in S. Pullorum
Source: PLoS One. 2018 Jul 20;13(7):e0200585. doi: 10.1371/journal.pone.0200585 (PMC6054384; doi:10.1371/journal.pone.0200585)
Supplement: S5 File — (PDF) [file pone.0200585.s008.pdf]

S5 File. Alignment of *ccmH*(2) CDS from *S*. Gallinarum strains 287/91 (SG287_91) and 9184 (SG9184), and *S*. Pullorum strains CDC1983-67 (SPCDC) and RKS5078 (SPRKS).

```
                                           20                                      40
SG287_91_ccmH(2)  ATGAGACTGTTACCGGGCATGGTGATGCTGATGCTGGTGCTGGTTATCTCCGGGTCA 57
  SG9184_ccmH(2)  ATGAGACTGTTACCGGGCATGGTGATGCTGATGCTGGTGCTGGTTATCTCCGGGTCA 57
   SPCDC_ccmH(2)  ATGAGACTGTTACCGGGCATGGTGATGCTGATGCTGGTGCTGGTTATCTCCGGGTCA 57
   SPRKS_ccmH(2)  ATGAGACTGTTACCGGGCATGGTGATGCTGATGCTGGTGCTGGTTATCTCCGGGTCA 57

                     60                                      80                                     100
SG287_91_ccmH(2)  GCGCGGGCGACCACCGACGTGATGCCGTTTAAAGATGAAGCGCAGGAGCAGCAGTTC 114
  SG9184_ccmH(2)  GCGCGGGCGACCACCGACGTGATGCCGTTTAAAGATGAAGCGCAGGAGCAGCAGTTC 114
   SPCDC_ccmH(2)  GCGCGGGCGACCACCGACGTGATGCCGTTTAAAGATGAAGCGCAGGAGCAGCAGTTC 114
   SPRKS_ccmH(2)  GCGCGGGCGACCACCGACGTGATGCCGTTTAAAGATGAAGCGCAGGAGCAGCAGTTC 114

                        120                                     140                                     160
SG287_91_ccmH(2)  CGCCAGCTCACGGAGCAGCTGCGCTGCCCGAAATGCCAGAACAACAGCATTGCGGAC 171
  SG9184_ccmH(2)  CGCCAGCTCACGGAGCAGCTGCGCTGCCCGAAATGCCAGAACAACAGCATTGCGGAC 171
   SPCDC_ccmH(2)  CGCCAGCTCACGGAGCAGCTGCGCTGCCCGAAATGCCAGAACAACAGCATTGCGGAC 171
   SPRKS_ccmH(2)  CGCCAGCTCACGGAGCAGCTGCGCTGCCCGAAATGCCAGAACAACAGCATTGCGGAC 171

                           180                                     200                                     220
SG287_91_ccmH(2)  TCGAACGCGATGATAGCCACCGACATGCGCCGCAGGGTGTATGACCTGATGCAGGAG 228
  SG9184_ccmH(2)  TCGAACGCGATGATAGCCACCGACATGCGCCGCAGGGTGTATGACCTGATGCAGGAG 228
   SPCDC_ccmH(2)  TCGAACGCGATGATAGCCACCGACATGCGCCGCAGGGTGTATGACCTGATGCAGGAG 228
   SPRKS_ccmH(2)  TCGAACGCGATGATAGCCACCGACATGCGCCGCAGGGTGTATGACCTGATGCAGGAG 228

                              240                                     260                                     280
SG287_91_ccmH(2)  GGGAAGAGCCGCCAGGAAATCATCGATTACATGGTGGCGCGCTACGGCAACTTCGTC 285
  SG9184_ccmH(2)  GGGAAGAGCCGCCAGGAAATCATCGATTACATGGTGGCGCGCTACGGCAACTTCGTC 285
   SPCDC_ccmH(2)  GGGAAGAGCCGCCAGGAAATCATCGATTACATGGTGGCGCGCTACGGCAACTTCGTC 285
   SPRKS_ccmH(2)  GGGAAGAGCCGCCAGGAAATCATCGATTACATGGTGGCGCGCTACGGCAACTTCGTC 285

                                 300                                     320                                     340
SG287_91_ccmH(2)  ACCTACGACCCGCCGCTGACCCCGCTGACCCCGCTGACGGTGCTGCTGTGGGTGCTG 342
  SG9184_ccmH(2)  ACCTACGACCCGCCGCTGACCCCGCTGACCCCGCTGACGGTGCTGCTGTGGGTGCTG 342
   SPCDC_ccmH(2)  ACCTACGACCCGCCGCTGACCCCGCTGAC---------GGTGCTGCTGTGGGTGCTG 333
   SPRKS_ccmH(2)  ACCTACGACCCGCCGCTGACCCCGCTGAC---------GGTGCTGCTGTGGGTGCTG 333

                                    360                                     380
SG287_91_ccmH(2)  CCGCTGGCCGCCATCGTGGCGGGCGGGTGGATAATCGTTGCCCGCACGCGCCGGCGG 399
  SG9184_ccmH(2)  CCGCTGGCCGCCATCGTGGCGGGCGGGTGGATAATCGTTGCCCGCACGCGCCGGCGG 399
   SPCDC_ccmH(2)  CCGCTGGCCGCTATCGTGGCGGGCGGGTGGATAATCGTTGCCCGCACGCGCCGGCGG 390
   SPRKS_ccmH(2)  CCGCTGGCCGCTATCGTGGCGGGCGGGTGGATAATCGTTGCCCGCACGCGCCGGCGG 390

                  400                                     420                                     440
SG287_91_ccmH(2)  GTGCGCCTGCGCCGGGAGCCGCTGCCGGCGGACACCCCGGTTTGCGGCGCGCGCGCC 456
  SG9184_ccmH(2)  GTGCGCCTGCGCCGGGAGCCGCTGCCGGCGGACACCCCGGTTTGCGGCGCGCGCGCC 456
   SPCDC_ccmH(2)  GTGCGCCTGCGCCGG------------------------------------------ 405
   SPRKS_ccmH(2)  GTGCGCCTGCGCCGG------------------------------------------ 405

                     460                                     480                                     500
SG287_91_ccmH(2)  GGGTGGGGCGTTTACGTGCCGGGGGCCGTCATTGCGCTGGCGGTCGGCGCCGGCAGC 513
  SG9184_ccmH(2)  GGGTGGGGCGTTTACGTGCCGGGGGCCGTCATTGCGCTGGCGGTCGGCGCCGGCAGC 513
   SPCDC_ccmH(2)  -----------------------------------------------------CAGC 409
   SPRKS_ccmH(2)  -----------------------------------------------------CAGC 409

                        520                                     540                                     560
SG287_91_ccmH(2)  TACGCCCTGACCGGCAGCTATCAGCAGGTCAGGGCCTGGCAGCAGGCAACGGCGCAG 570
  SG9184_ccmH(2)  TACGCCCTGACCGGCAGCTATCAGCAGGTCAGGGCCTGGCAGCAGGCAACGGCGCAG 570
   SPCDC_ccmH(2)  TACGCCCTGACCGGCAGCTATCAGCAGGTCAGGGCCTGGCAGCAGGCAACGGCGCAG 466
   SPRKS_ccmH(2)  TACGCCCTGACCGGCAGCTATCAGCAGGTCAGGGCCTGGCAGCAGGCAACGGCGCAG 466
```

```
                                       580                                        600                                       620
SG287_91_ccmH(2)  ACGCCCGGGCTGCTGGCGCGGGCGCTGGACCCGGCGGCGCAGCCGCTGAATGAAGAG 627
SG9184_ccmH(2)    ACGCCCGGGCTGCTGGCGCGGGCGCTGGACCCGGCGGCGCAGCCGCTGAATGAAGAG 627
SPCDC_ccmH(2)     ACGCCCGGGCTGCTGGCGCGGGCGCTGGACCCGGCGGCGCAGCCGCTGAATGAAGAG 523
SPRKS_ccmH(2)     ACGCCCGGGCTGCTGGCGCGGGCGCTGGACCCGGCGGCGCAGCCGCTGAATGAAGAG 523
                                        640                                        660                                       680
SG287_91_ccmH(2)  GAGATGGCGCGGCTGGCGCTGGGGCTGCGCACCCGCCTGCAGAATGATGCCGGCAAT 684
SG9184_ccmH(2)    GAGATGGCGCGGCTGGCGCTGGGGCTGCGCACCCGCCTGCAGAATGATGCCGGCAAT 684
SPCDC_ccmH(2)     GAGATGGCGCGGCTGGCGCTGGGGCTGCGCACCCGCCTGCAGAATGATGCCGGCAAT 580
SPRKS_ccmH(2)     GAGATGGCGCGGCTGGCGCTGGGGCTGCGCACCCGCCTGCAGAATGATGCCGGCAAT 580
                                           700                                        720                                       740
SG287_91_ccmH(2)  GTTGAGGGCTGGCTCATGCTGGGG-CGCACCGGTATGGTACTGGGTAATGCCGGTAC 740
SG9184_ccmH(2)    GTTGAGGGCTGGCTCATGCTGGGG-CGCACCGGTATGGTACTGGGTAATGCCGGTAC 740
SPCDC_ccmH(2)     GTTGAGGGCTGGCTCATGCTGGGGGCGCACCGGTATGGTACTGGGTAATGCCGGTAC 637
SPRKS_ccmH(2)     GTTGAGGGCTGGCTCATGCTGGGGGCGCACCGGTATGGTACTGGGTAATGCCGGTAC 637
                                              760                                        780
SG287_91_ccmH(2)  CGCCACCGGGGCCTATGCGAACGCCTACCGCCTGGACCCGAAAAACAGCGATGCGGC 797
SG9184_ccmH(2)    CGCCACCGGGGCCTATGCGAACGCCTACCGCCTGGACCCGAAAAACAGCGATGCGGC 797
SPCDC_ccmH(2)     CGCCACCGGGGCCTATGCGAACGCCTACCGCCTGGACCCGAAAAACAGCGATGCGGC 694
SPRKS_ccmH(2)     CGCCACCGGGGCCTATGCGAACGCCTACCGCCTGGACCCGAAAAACAGCGATGCGGC 694
                    800                                        820                                        840
SG287_91_ccmH(2)  GCTGGGCTACGCGGAGGCGCTGACGCGCTCGTCCGACCCGGAGGATAACCGCGGCGG 854
SG9184_ccmH(2)    GCTGGGCTACGCGGAGGCGCTGACGCGCTCGTCCGACCCGGAGGATAACCGCGGCGG 854
SPCDC_ccmH(2)     GCTGGGCTACGCGGAGGCGCTGACGCGCTCGTCCGACCCGGAGGATAACCGCCGCGG 751
SPRKS_ccmH(2)     GCTGGGCTACGCGGAGGCGCTGACGCGCTCGTCCGACCCGGAGGATAACCGCCGCGG 751
                       860                                        880                                        900
SG287_91_ccmH(2)  CGGGGAGCTGCTGCGTCAGCTGGTGAGAAGTGACCACACGGATATCCGGGTGTTAAG 911
SG9184_ccmH(2)    CGGGGAGCTGCTGCGTCAGCTGGTGAGAAGTGACCACACGGATATCCGGGTGTTAAG 911
SPCDC_ccmH(2)     CGGGGAGCTGCTGCGTCAGCTGGTGAGAAGTGACCACACGGATATCCGGGTGTTAAG 808
SPRKS_ccmH(2)     CGGGGAGCTGCTGCGTCAGCTGGTGAGAAGTGACCACACGGATATCCGGGTGTTAAG 808
                          920                                        940                                        960
SG287_91_ccmH(2)  CCTGTATGCGTTCAGCGCCTTTGAGCAGCAGCGTTTTGGCGAGGCGGTGGCGGCCTG 968
SG9184_ccmH(2)    CCTGTATGCGTTCAGCGCCTTTGAGCAGCAGCGTTTTGGCGAGGCGGTGGCGGCCTG 968
SPCDC_ccmH(2)     CCTGTATGCGTTCAGCGCCTTTGAGCAGCAGCGTTTTGGCGAGGCGGTGGCGGCCTG 865
SPRKS_ccmH(2)     CCTGTATGCGTTCAGCGCCTTTGAGCAGCAGCGTTTTGGCGAGGCGGTGGCGGCCTG 865
                             980                                      1.000                                      1.020
SG287_91_ccmH(2)  GGAGATGATGCTGAAACTGCTGCCGGCGGGTGACGCCCGGCGGGCGGTGATAGAGCG 1025
SG9184_ccmH(2)    GGAGATGATGCTGAAACTGCTGCCGGCGGGTGACGCCCGGCGGGCGGTGATAGAGCG 1025
SPCDC_ccmH(2)     GGAGATGATGCTGAAACTGCTGCCGGCGGGTGACGCCCGGCGGGCGGTGATAGAGCG 922
SPRKS_ccmH(2)     GGAGATGATGCTGAAACTGCTGCCGGCGGGTGACGCCCGGCGGGCGGTGATAGAGCG 922
                                1.040
SG287_91_ccmH(2)  CAGTATCCGGCTGGCGCAGGAGAAATAA 1053
SG9184_ccmH(2)    CAGTATCCGGCTGGCGCAGGAGAAATAA 1053
SPCDC_ccmH(2)     CAGTATCCGGCTGGCGCAGGAGAAATAA 950
SPRKS_ccmH(2)     CAGTATCCGGCTGGCGCAGGAGAAATAA 950
```
